# Human Cytomegalovirus Latency-Associated Proteins Elicit Immune-Suppressive IL-10 Producing CD4^+^ T Cells

**DOI:** 10.1371/journal.ppat.1003635

**Published:** 2013-10-10

**Authors:** Gavin M. Mason, Sarah Jackson, Georgina Okecha, Emma Poole, J. G. Patrick Sissons, John Sinclair, Mark R. Wills

**Affiliations:** University of Cambridge, Department of Medicine, Cambridge, Cambridgeshire, United Kingdom; Baylor College of Medicine, United States of America

## Abstract

Human cytomegalovirus (HCMV) is a widely prevalent human herpesvirus, which, after primary infection, persists in the host for life. In healthy individuals, the virus is well controlled by the HCMV-specific T cell response. A key feature of this persistence, in the face of a normally robust host immune response, is the establishment of viral latency. In contrast to lytic infection, which is characterised by extensive viral gene expression and virus production, long-term latency in cells of the myeloid lineage is characterised by highly restricted expression of viral genes, including UL138 and LUNA. Here we report that both UL138 and LUNA-specific T cells were detectable directly *ex vivo* in healthy HCMV seropositive subjects and that this response is principally CD4^+^ T cell mediated. These UL138-specific CD4^+^ T cells are able to mediate MHC class II restricted cytotoxicity and, importantly, show IFNγ effector function in the context of both lytic and latent infection. Furthermore, in contrast to CD4^+^ T cells specific to antigens expressed solely during lytic infection, both the UL138 and LUNA-specific CD4^+^ T cell responses included CD4^+^ T cells that secreted the immunosuppressive cytokine cIL-10. We also show that cIL-10 expressing CD4^+^ T-cells are directed against latently expressed US28 and UL111A. Taken together, our data show that latency-associated gene products of HCMV generate CD4^+^ T cell responses *in vivo*, which are able to elicit effector function in response to both lytic and latently infected cells. Importantly and in contrast to CD4^+^ T cell populations, which recognise antigens solely expressed during lytic infection, include a subset of cells that secrete the immunosuppressive cytokine cIL-10. This suggests that HCMV skews the T cell responses to latency-associated antigens to one that is overall suppressive in order to sustain latent carriage *in vivo*.

## Author Summary

Human cytomegalovirus (HCMV) is a widely prevalent virus, which is normally carried without clinical symptoms, but often causes severe clinical disease in individuals with compromised immune responses. In healthy HCMV carriers, the immune response to HCMV is robust and includes large numbers of virus-specific T-cells that control viral replication during active infection. Despite this prodigious immune response, HCMV is never cleared after primary infection but persists in the host for life: a key feature of persistence is the ability of the virus to establish a type of viral quiescence, termed latency. Although much is known about T-cell responses to viral proteins expressed solely during lytic infection, the interplay between the T-cell response and latent HCMV is not well understood. Here we report the first comprehensive characterisation of the T-cell response to latent HCMV and show that it is composed principally of CD4^+^ T-cells, which are specific for viral proteins expressed during latency, and are able to detect latent virus *in vitro*. We further show that these CD4^+^ T-cell responses to latency-associated viral gene products include T-cells that secrete cIL-10, an immunosuppressive cytokine, which may function to suppress antiviral immune responses and thereby maintain lifelong carriage of the latent virus.

## Introduction

Human cytomegalovirus (HCMV) is widely prevalent, with an estimated 50–60% of the world population being seropositive [1]. Primary infection in the immunocompetent host is usually asymptomatic and overt disease is seen almost exclusively in the immunocompromised and immuno-naive host. For example, placental transmission of HCMV is a leading infective cause of congenital abnormalities [2]. Primary infection with HCMV induces a robust innate and adaptive immune response, which includes a substantial CD4^+^ and CD8^+^ T cell response [2]–[4], which is essential for the control of HCMV disease [5]–[8]. However, despite this extensive immune response, HCMV is not cleared but persists for the lifetime of the host due, at least in part, to the establishment of viral latency in certain cell types, where the viral genome is carried in the absence of the production of infectious viral progeny [9], [10].

One defined site of latency of HCMV *in vivo* is in cells of the myeloid lineage, including CD34^+^ haematopoietic progenitor cells of the bone marrow [11]–[14]. Furthermore, differentiation of CD34^+^ cells to terminally differentiated cells of the myeloid lineage, such as macrophages or dendritic cells, results in reactivation of infectious virus [12], [15]–[17]. Despite probable frequent occurrences of reactivation events *in vivo*, these events are likely asymptomatic due to an active and robust immune response. In contrast, in an immunocompromised setting, the lack of a functional T cell response results in uncontrolled virus replication, which can occur during primary infection, super-infection or reactivation [2], [18], [19] and result in significant clinical disease [2], [18].

The viral proteins recognised by HCMV-specific T cells during lytic infection have been extensively investigated. The immediate early proteins IE1 and IE2 (UL123 and UL122, respectively), as well as the tegument protein pp65 (UL83), were all recognized by both CD4^+^ and CD8^+^ T cells in the majority of individuals, regardless of HLA type. In contrast, although T cells specific for glycoprotein B (UL55) are also frequently generated they are predominantly CD4^+^ T cells [20]–[23]. Using synthetic peptide libraries spanning the entire predicted HCMV proteome, a comprehensive analysis of the breadth and frequency of the CD4^+^ and CD8^+^ T cell response to HCMV has been carried out. These data showed that, in a large cohort of healthy seropositive donors with a diverse range of HLA types, a CD4^+^ or CD8^+^ T cell response was detectable to over 150 viral ORFs which included both structural and non-structural proteins expressed during all phases of lytic infection [20], [21]. Furthermore, they also observed that in any given donor CD4^+^ and CD8^+^ T cell responses recognised a median of 8 ORFs and 12 ORFs, respectively, suggesting that a large repertoire of HCMV ORFs were recognised by the immune response.

In contrast to lytic infection, during which pivotal genes such as the viral immediate early (IE) genes drive expression of early and late genes such as pp65 and gB, viral gene expression during latency is highly restricted. In the absence of IE gene expression, an accepted characteristic of latent infection, only a handful of viral genes have been shown to be expressed during natural and experimental models of latent infection [24]–[31]. These include: UL138, LUNA (latency-associated unidentified nuclear antigen) an antisense transcript to the UL81–82 region, UL111A (vIL-10) a viral homologue of cellular IL-10 (cIL-10) and US28, a chemokine receptor homologue [28], [32].

Although the functional role of these latency-associated genes during latency is far from clear, we hypothesised that the robust and antigenically broad T cell immune response elicited by HCMV would likely include responses to these latency-associated antigens, since they are expressed during lytic infection also. Consistent with this, recent evidence has suggested a limited CD8^+^ T cell response to UL138 is present in healthy individuals [33]. However, if this was generally the case it would raise the question as to why such responses do not expose the latently infected cell to immune recognition and eventual clearance. Clearly, understanding this is of crucial importance to understanding the mechanism of latent carriage.

In this study we have measured and characterised UL138 and LUNA specific T cell responses in a cohort of healthy HCMV seropositive donors. Here we show, that they elicit predominantly CD4^+^ T_h1_ type responses, characterised by IFNγ production. Additionally, we observe that UL138 specific CD4^+^ T cells mediated MHC class II restricted cytotoxicity. These CD4^+^ T cells are able to recognize antigen both in lytically infected dendritic cells and, importantly, they also recognize latently infected monocytes. Intriguingly, a proportion of the UL138 and LUNA specific CD4^+^ T cells also secreted the immunomodulatory cytokines cIL10 and transforming growth factor β (TGF-β). Importantly, additional analysis of two other latency-associated proteins (US28 and vIL-10), showed that these antigens also generated CD4^+^ T cell responses which again were able to secrete cIL-10. To our knowledge this is the first description of HCMV specific cIL-10 producing CD4^+^ T cells in normal healthy HCMV seropositive individuals. We hypothesise that this T cell response, which includes potentially suppressive T cells specific to latent antigens, may function to assist in the lifelong carriage and maintenance of the latent reservoir of infection by preventing efficient T_h1_ T cell recognition of latently infected cells.

## Results

### UL138 and LUNA specific T cells are generated by HCMV seropositive individuals

In order to determine whether T cell responses to UL138 or LUNA proteins could be detected, IFNγ specific ELISPOT assays were performed on whole freshly isolated peripheral blood mononuclear cells (PBMC) or PBMC depleted of either CD4^+^ or CD8^+^ T cells from both HCMV seropositive (n = 17) and seronegative donors (n = 6). Isolated cells were stimulated with overlapping pools of peptides spanning either the UL138 or LUNA predicted open reading frames and T cell recognition was determined by IFNγ production. In parallel, we performed a concomitant analysis of the response to pools of peptides spanning the HCMV ORFs IE1/2 (UL123 and UL122), pp65 (UL83) and gB (UL55), which are well defined CD4^+^ and CD8^+^ T cell antigens in HCMV seropositive individuals (Table 1).

**Table 1:** Summary of HCMV specific IFNγ secreting T cell response.

Donor	pp65			IE			gB			UL138			LUNA		
	PBMC	CD4	CD8	PBMC	CD4	CD8	PBMC	CD4	CD8	PBMC	CD4	CD8	PBMC	CD4	CD8
**300**	2500	720	2500	2448	1226	2500	2500	2500	94	524	1295	5	360	195	0
**301**	1800	825	2500	2500	2500	2146	2500	1360	24	120	168	8	138	125	15
**302**	2500	624	2500	2500	1284	2500	1682	1842	0	15	20	20	180	100	24
**303**	565	200	840	682	648	702	1342	1120	0	0	0	0	0	0	0
**304**	1640	0	1840	1080	42	1246	80	64	0	0	4	8	120	104	24
**305**	556	256	0	2328	2024	1462	2500	2500	642	656	1161	2	32	11	14
**306**	120	0	0	240	0	182	1340	1284	0	20	5	5	60	16	4
**307**	400	0	542	1650	4	1280	0	0	0	0	0	0	0	0	0
**308**	410	665	295	1274	24	1426	440	526	0	20	0	10	240	132	32
**309**	1240	0	874	1862	60	1642	148	206	0	0	4	4	0	4	4
**312**	642	128	536	2500	2242	2500	78	24	0	20	8	4	0	0	4
**314**	648	0	438	750	0	422	42	86	0	0	0	0	137	148	0
**315**	2236	246	2214	2026	466	2500	642	588	226	0	0	0	0	0	0
**316**	2248	2500	2500	2500	2500	1240	2500	2500	680	680	520	12	460	270	44
**317**	468	324	120	2500	642	2238	2500	2500	0	227	120	4	426	446	0
**318**	824	128	642	2500	804	2500	0	0	0	24	0	4	62	8	4
**319**	628	94	680	2242	1600	2208	1862	2234	0	0	12	8	0	8	4

Whole PBMC or PBMC depleted of either CD4^+^ or CD8^+^ T cells from 17 HCMV seropositive donors were stimulated with overlapping peptide pools spanning the HCMV ORFs pp65, IE, gB, UL138 and LUNA. Cells were incubated for 48 hours and IFNγ production was detected by ELISPOT. Post incubation IFNγ spot forming units (SFU) were enumerated and background levels of IFNγ determined from a non-peptide stimulated control were subtracted from each value and converted into SFU/10^6^ cells.

As expected, substantial spot forming units (SFU) (SFU/10^6^ >100) CD4^+^ and CD8^+^ T cell responses to pp65 (CD8^+^ T cells 15/17 donors and CD4^+^ T cells 12/17 donors), IE (CD8^+^ T cells 17/17 and CD4^+^ T cells 11/17 donors) and gB (CD8^+^ T cells 3/17 and CD4^+^ T cells 12/17 donors) were readily detected in the HCMV seropositive, but not seronegative donors.

Interestingly, T cell responses to UL138 and LUNA were also detected in many of the donors tested with responses greater than 100 SFU/10^6^ cells to UL138 in 5/17 donors (range 120–1295 SFU/10^6^ cells) and to LUNA in 8/17 donors (range 100–446 SFU/10^6^ cells) seropositive donors tested. Furthermore, responses >100 SFU/10^6^ cells were mediated exclusively by CD4^+^ T cells in all the UL138 and LUNA reactive donors (Table 1); none of the seronegative donors tested made responses (data not shown).

We confirmed that all donor PBMC and CD4/CD8 –depleted PBMCs tested retained IFNγ producing effector function following polyclonal stimulation using phytohaemagglutinin (PHA) (data not shown). Taken together, these results clearly show that both UL138 and LUNA generate a CD4^+^ T cell response and, furthermore, the frequency of the response was in the lower to middle range compared to CD4^+^ T cell responses to gB (Table 1). In order to expand any UL138 specific T cells that were below the detection limit of the ex-vivo ELISPOT assays purified CD8^+^ T cells from three different donors were stimulated with the UL138 peptide pool and cultured for 12 days. These cultures were then retested for UL138 specific responses by ELISPOT assays and were negative in all cases (data not shown).

In order to confirm the frequencies of these CD4^+^ T cell responses as well as determine more precisely the epitope specificity, individual 15 amino acid overlapping peptides that composed the UL138 and LUNA ORF pools were tested for T cell reactivity against each donor. All 17 HCMV seropositive donors were re-tested using the individual peptides from each ORF, irrespective of whether they had a detectable response to UL138 or LUNA ORF pools in the initial screen.

**Figure 1:**
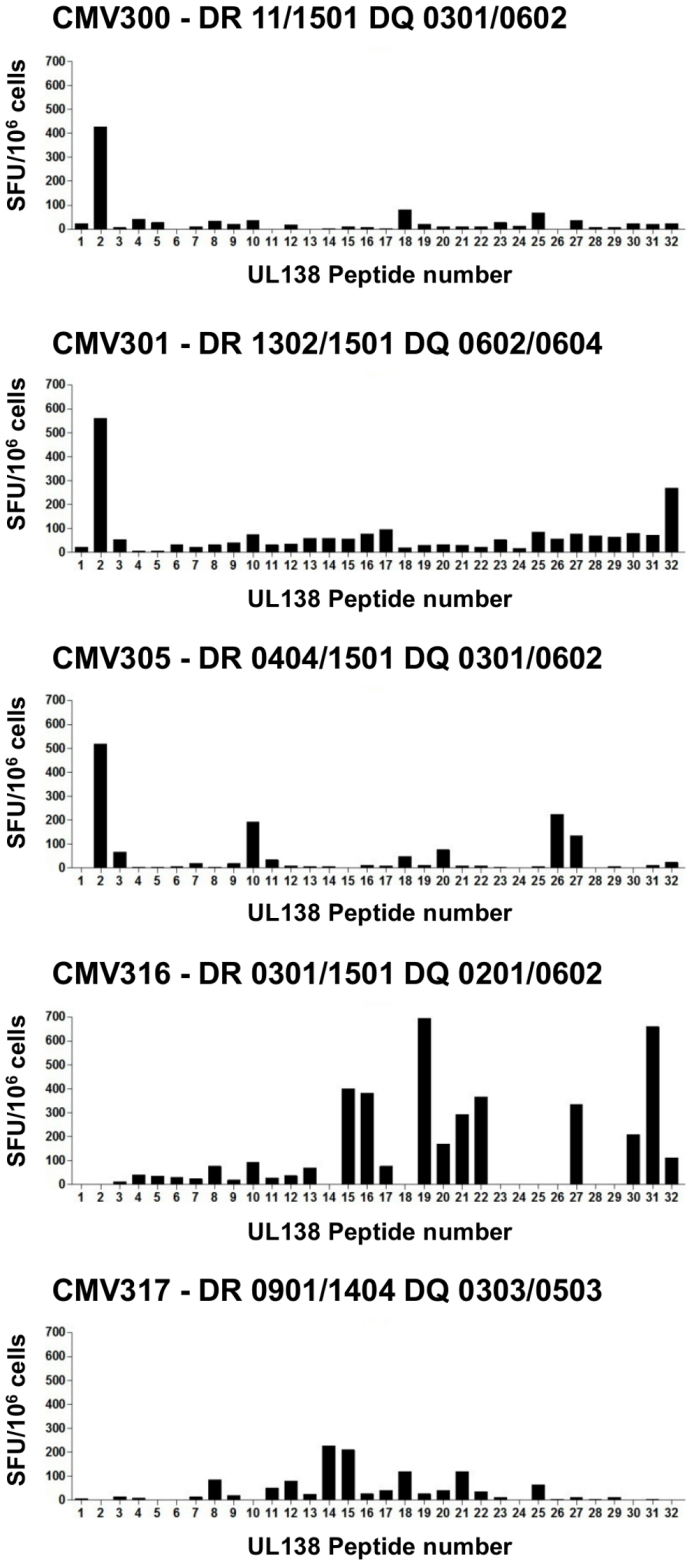
Mapping of UL138 specific T cell responses to individual 15 amino acid peptides. PBMC from 17 HCMV seropositive donors were stimulated with 32 individual 15 amino acid overlapping peptides covering UL138 ORF in a 48γ ELISPOT assay. Post incubation IFNγ spot forming units (SFU) were enumerated and the value from an unstimulated control was subtracted before conversion to SFU/10^6^ cells. Data shown is from the 5 donors who made T cell responses to the complete UL138 ORF peptide pool (Table 1).

**Figure 2:**
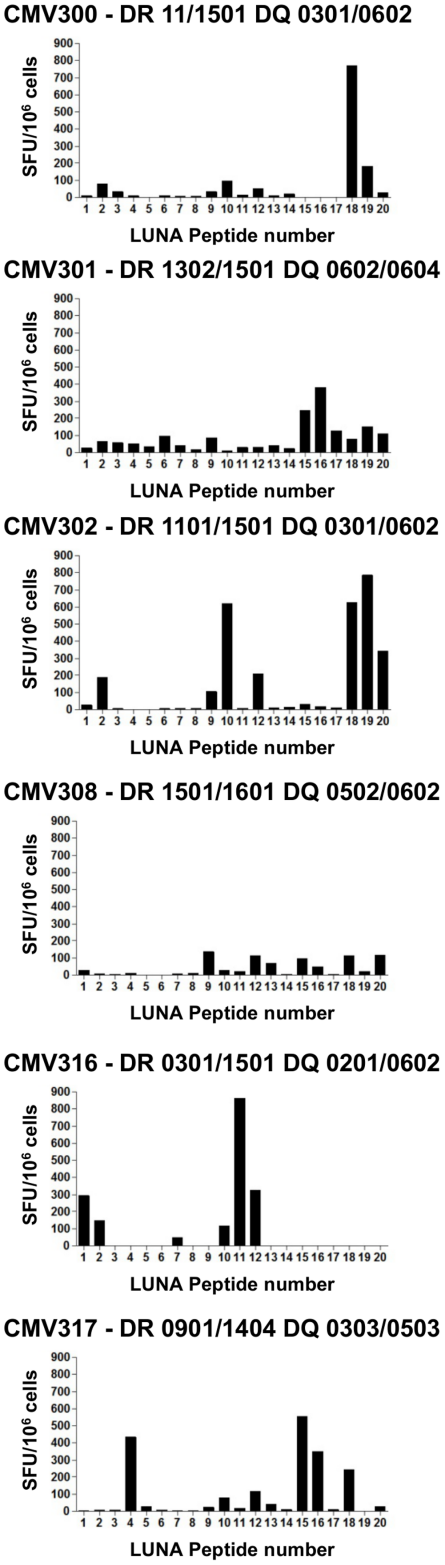
Mapping of LUNA specific T cell responses to individual 15 amino acid peptides. PBMC from 17 HCMV seropositive donors were stimulated with 20 individual 15amino acid overlapping peptides covering the LUNA peptide pool in a 48γ ELISPOT assay. Post incubation IFNγ spot forming units (SFU) were enumerated and the value from an unstimulated control was subtracted before conversion to SFU/10^6^ cells. Data shown is from the 6 donors who made T cell responses to the whole LUNA ORF peptide pool (Table 1).

The results show that no additional UL138 or LUNA IFNγ secreting T cell responses were detected by stimulation with the individual peptides. All five donors previously shown to elicit a CD4^+^ T cell responses to the UL138 ORF pool were restricted to an immunogenic 15mer peptide or clusters of overlapping peptides (Figure 1) including 3 donors (CMV300, 301, and 305) who elicited high level IFNγ production to the same 15mer peptide; UL138 peptide 2 (LNVGLPIIGVMLVLI). Similarly, six of the seven donors with a detectable LUNA specific T cell response were mapped to either a 15mer peptide or alternatively to a clusters of overlapping 15mer peptides (Figure 2). Again 3 donors (CMV300, 302 and 317) showed IFNγ^+^ T cell responses to the same 15mer peptide, LUNA peptide 18 (RLILSGLPGVRVQNP).

### UL138 and LUNA specific CD4^+^ T cell lines recognise lytic HCMV infection of monocyte derived dendritic cells

**Figure 3:**
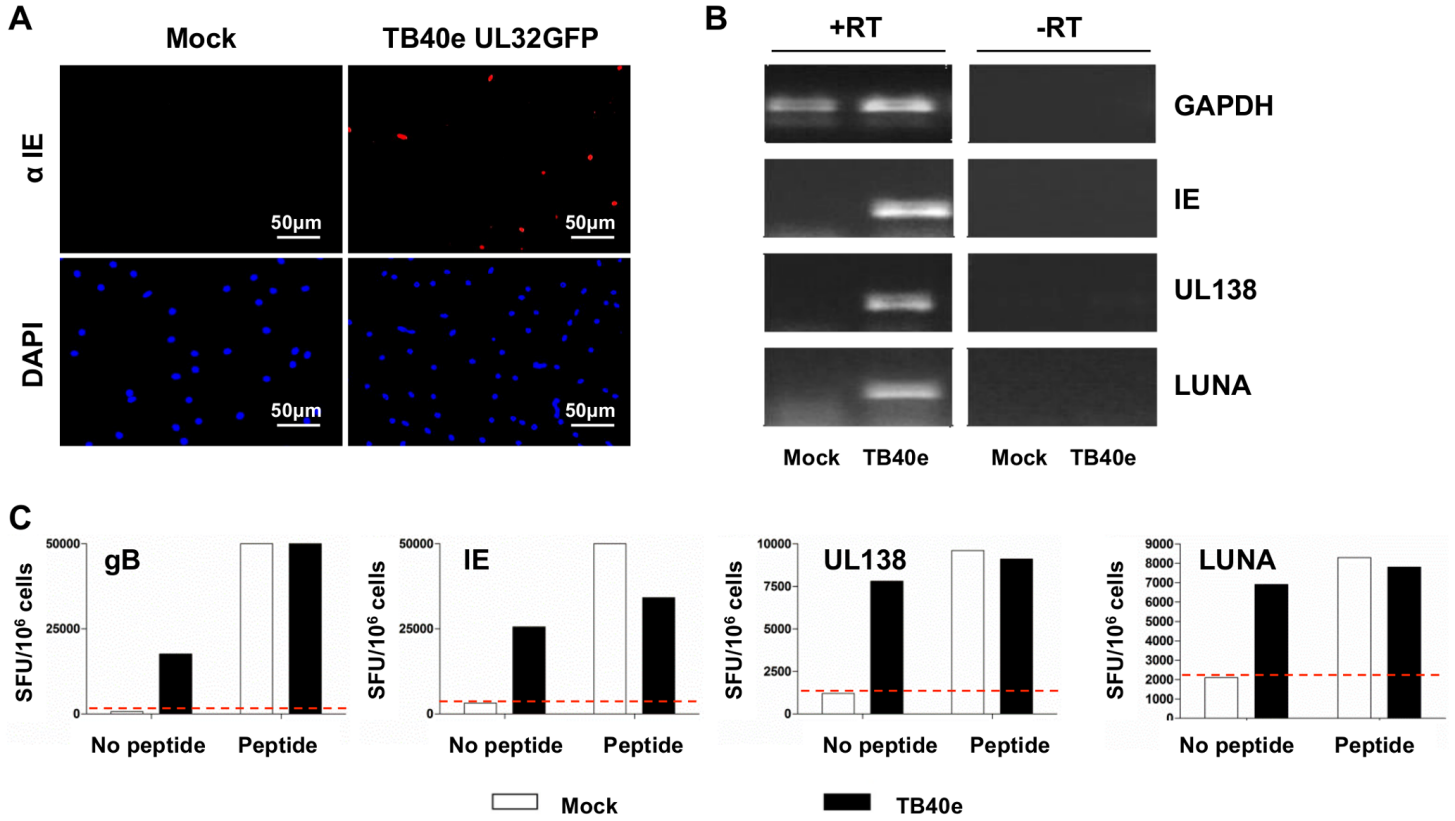
UL138 and LUNA specific CD4^+^ T cells are stimulated by HCMV lytic infection of monocyte derived dendritic cells. Dendritic cells were prepared from donor CMV300 and mock or lytically infected with HCMV strain TB40e at MOI 5. After 5 days lytic infection was confirmed by IE expression using immunofluorescence (A) and RT-PCR (B). Autologous mock or TB40e infected dendritic cells were then co-incubated with *in vitro* expanded antigen specific CD4^+^ T cells specific to gB, IE, UL138 or LUNA in IFNγ ELISPOT assays in the presence or absence of cognate peptide (C). Post incubation IFNγ spot forming units (SFU/10^6^) were enumerated and the back ground level of IFNγ production for each antigen specificity determined from the mock infected no peptide control (Red dotted line).

UL138 and LUNA specific T cells are clearly generated in response to HCMV infection and can be re-stimulated with synthetic peptides. Consequently, we next addressed whether UL138 and LUNA specific CD4^+^ T cells could be activated in response to cells lytically infected with HCMV. CD4^+^ T cell lines specific for UL138, LUNA as well as gB and IE were incubated with either HCMV infected autologous monocyte derived dendritic cells (moDC) or mock infected cells (Phenotypic analysis of moDC shown in Figure S1). Following infection with HCMV, approximately 10% of moDC cells were IE positive (Figure 3A). Lack of availability of antibodies against UL138 and LUNA meant an RT-PCR analysis was performed instead. Using this approach we confirmed the expression of IE, UL138 and LUNA mRNAs expression in lytically infected moDCs (Figure 3B). As expected, all samples were positive for GAPDH and mock-infected moDCs expressed no viral transcripts.

The results clearly show that both the UL138 and LUNA specific T cells from donor CMV300 were activated following stimulation with lytically infected moDC (Figure 3C). As expected, gB and IE specific CD4^+^ T cells were also stimulated by virus infected cells (Figure 3C). Consistent with specificity, all CD4^+^ T cell lines were activated by the relevant gB, IE, UL138 and LUNA peptide pulsed moDC. In contrast, no IFNγ was detected following stimulation with uninfected/peptide untreated moDC. Similarly, CD4^+^ T cells specific for UL138 from a second donor (CMV305) were tested for recognition of HCMV infected autologous moDC. Again, we observed activation of all three specificities in this donors' CD4^+^ T cells (Figure S2). Furthermore, repetition of the analyses on independent occasions confirmed that, from both donors, latent specific T cells were activated by lytically infected moDC (data not shown).

### UL138 specific CD4^+^ T cells recognise and secrete IFNγ in response to latently infected monocytes

**Figure 4:**
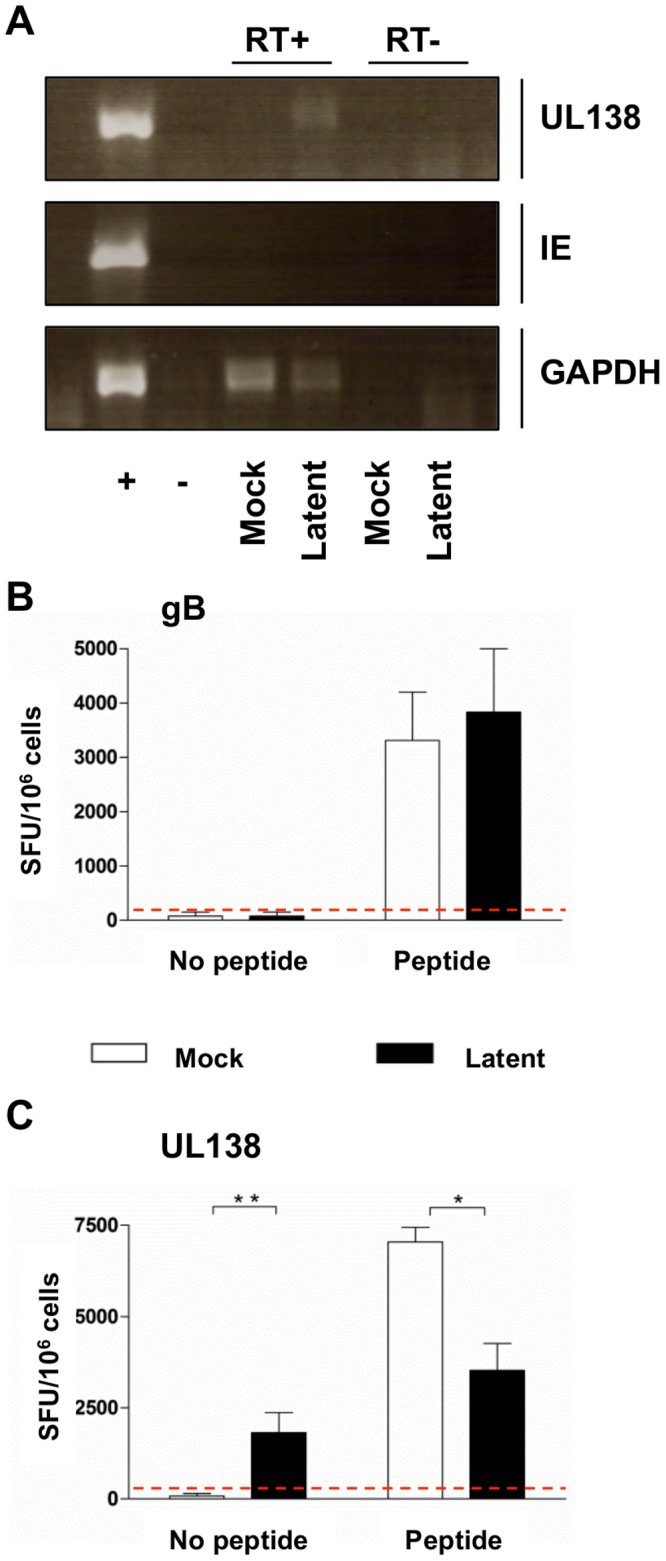
UL138 specific CD4^+^ T cells secrete IFNγ in response to latently infected monocytes. Monocytes were prepared from donor CMV305 and mock infected or latently infected with TB40e for 10 days at MOI 5. Latent infection was then confirmed by RT-PCR (A). Autologous mock or latently infected monocytes were then co-incubated with *in vitro* expanded antigen specific CD4^+^ T cells specific to gB (B) and UL138 (C) in IFNγ ELISPOT assays in the presence or absence of cognate peptide. Post incubation IFNγ spot forming units (SFU/10^6^) were enumerated and the back ground level of IFNγ production for each antigen specificity determined from the mock infected no peptide control (Red dotted line). Error bars are standard error of the mean (n = 5). Statistical analysis were performed using the students t test (* p<0.05;** p<0.01).

The previous experiments clearly demonstrated that HCMV lytic infection of MHC Class II positive dendritic cells lead to antigen presentation of both UL138 and LUNA peptides and subsequent CD4^+^ T cell recognition. An important question was whether these T cells could also be activated by latently infected autologous monocytes. In order to determine if UL138 specific CD4^+^ T cells could recognize latently infected cells, autologous monocytes were prepared and latently infected with HCMV strain TB40e for 10 days. The establishment of latency was confirmed by RT-PCR analysis; latently infected monocytes were shown to express the UL138 latent transcript with an absence of IE mRNA expression (Figure 4A). Mock-infected monocytes did not express UL138, while both mock and latently infected cells were GAPDH positive.

UL138 and gB specific CD4^+^ T cells from donor CMV305 were generated as previously described and co-incubated with autologous latently or mock infected monocytes in IFNγ ELISPOT assays. As expected, no gB specific CD4^+^ T cell response against HCMV latently infected monocytes was detected. In contrast, gB specific CD4^+^ T cells clearly produced IFNγ if the mock or latently infected monocytes had been pulsed with gB peptide (Figure 4B). These results are consistent with a lack of gB expression during latent infection of monocytes but confirm that failure to detect gB is not due to a defect in antigen presentation to gB-specific CD4+ T cells by autologous monocytes.

Interestingly, in contrast to our observations with gB, our results show that UL138 specific CD4^+^ T cells produce IFNγ in response to incubation with latently infected monocytes (Figure 4C) as well as when cells were pulsed with UL138 peptide. Identical analyses were also performed using donor CMV300, which confirmed the recognition of latently infected monocytes by CD4^+^ T cells specific to UL138 (Figure S3). Further validation was achieved with subsequent analyses of T cells and monocytes derived from donors CMV300 and CMV305 which, again, confirmed that UL138 specific T cells recognized HCMV latently infected monocytes (data not shown). To date, we have not been able to demonstrate if LUNA protein expression in latently infected cells can be recognised by LUNA specific T cells. Whether this is experimental failure or intrinsic to LUNA remains an open question and is currently under investigation.

### UL138 but not LUNA specific CD4^+^ T cells mediate MHC Class II restricted cytotoxicity

Classically, CD4^+^ T cells are considered helper T cells, exerting effector functions by cytokine secretion *in vivo*. Indeed, it is well established that T cell mediated cytotoxicity effector function is associated with CD8^+^ T cell recognition of antigen presented by MHC Class I. However, recent studies suggest that CD4^+^ T cells may play a more direct role in viral infection. Pertinent to this study, it has been shown in the context of HCMV infection that T_h1_ type gB specific CD4^+^ T cells that secrete IFNγ and TNFα have also been shown to be able to mediate MHC class II restricted cytotoxicity [21], [34]–[36].

Consequently, since both the UL138 and LUNA specific CD4^+^ T cell response is also characterised by IFNγ production, we next asked if these CD4^+^ T cells also have cytotoxic effector cell function. To do this, UL138 specific CD4^+^ T cell lines from donors CMV300 and CMV305 as well LUNA specific CD4^+^ T cell lines from CMV300 were expanded *in vitro* for two weeks and then tested for MHC Class II restricted cytotoxicity *in vitro* using chromium release assays. Again gB specific CD4^+^ T cells were used as a positive control.

**Figure 5:**
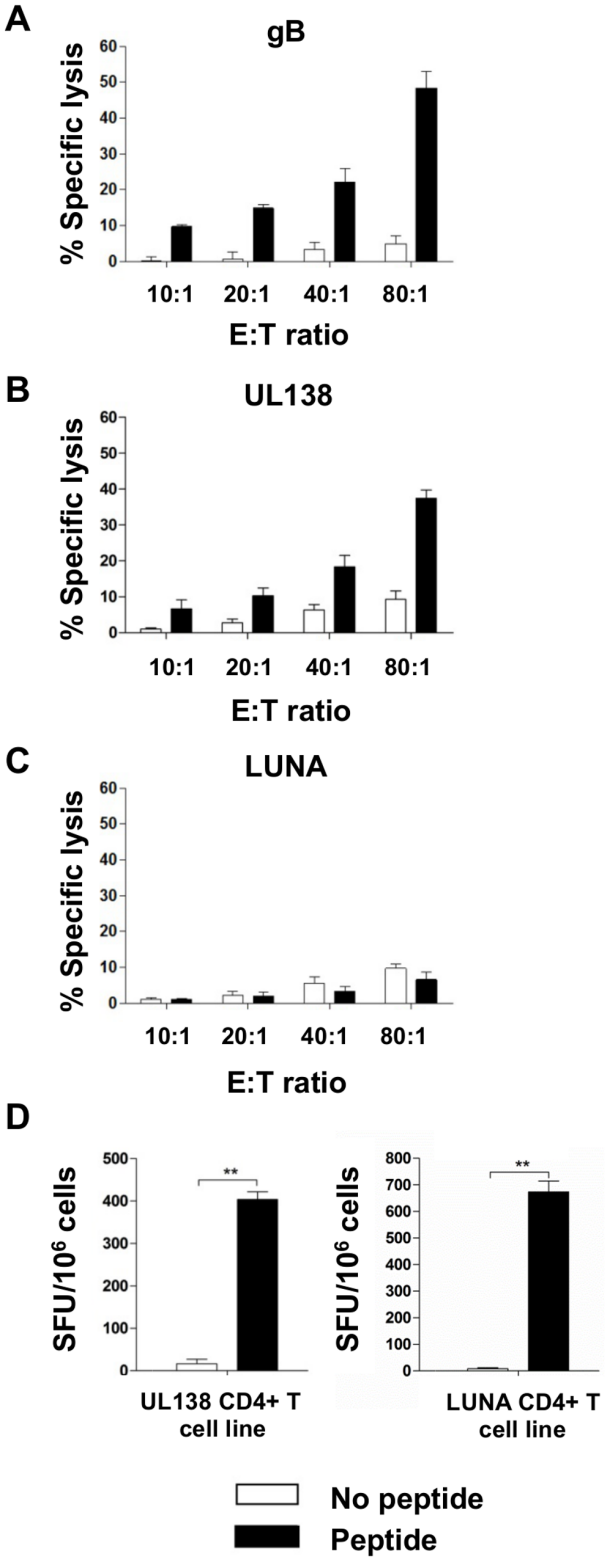
CD4^+^ T cells specific to UL138, but not LUNA can mediate MHC class II restricted cytotoxicity. Antigen specific CD4^+^ T cells specific to gB (A), UL138 (B) and LUNA (C) from donor CMV300 were expanded *in vitro* and incubated with autologous LCL in the presence or absence of cognate peptide at a range of effector to target cell ratios (E∶T) in 6 hour chromium release assays. Percent specific lysis was calculated to determine cytotoxic effector potential of the antigen specific T cells. Values >10% were deemed positive. Error bars are standard error of the mean (n = 6). The LUNA specific T cell line used in (C) was tested for specificity in IFNγ ELISPOT assays (D). Error bars represent standard error of the mean (n = 3). Statistical analysis was performed using the students t test (** p<0.01).

Consistent with previously published findings [34], [35] gB specific CD4^+^ T cells mediated cytotoxicity (Figure 5A). Furthermore, our results now show that UL138 specific CD4^+^ T cells are also able to mediate cytotoxicity (Figure 5B). In contrast, LUNA specific CD4^+^ T cells were not cytotoxic at any E∶T ratio examined (range 10∶1–80∶1) (Figure 5C), although importantly, the LUNA specific T cells remained antigen reactive by IFNγ specific ELISPOT assays (Figure 5D).

### UL138 and LUNA specific CD4^+^ T cells include both IFNγ and cIL-10 producing CD4^+^ T cells

The CD4^+^ T cell response is potentially composed of multiple subsets of CD4^+^ T cells with distinct functions and characteristic cytokines they produce. The HCMV specific CD4^+^ T cell response is characterised as being almost exclusively T_h1_ mediated, secreting IFNγ in response to lytic antigens such as gB and IE. However, it is interesting to note that parallels with other herpes viruses may be apparent: CD4^+^ T cells specific for a latent protein of EBV (LMP1) are able to secrete the immunosuppressive cytokine cIL-10 which is thought to play a role in evading immune responses during latent infection and maintenance of EBV latency [37]–[39]. Consequently, we analysed the cytokine profile of UL138 and LUNA specific CD4^+^ T cells following antigen stimulation and compared this to the well characterized response made by gB specific CD4^+^ T cells using a multi-analyte Th1/Th2 cytokine assay which measured 11 cytokines simultaneously.

**Figure 6:**
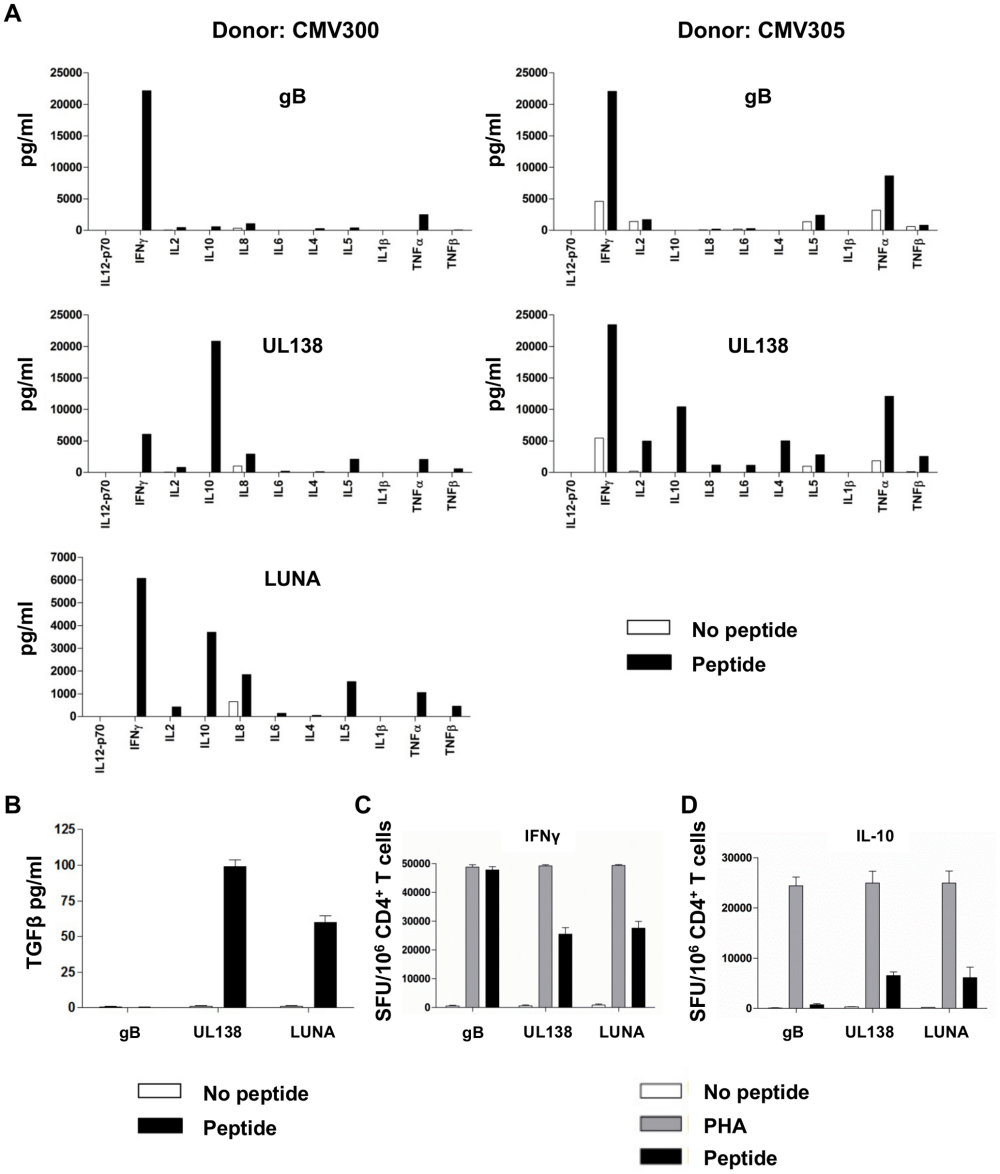
*In vitro* expanded UL138 and LUNA specific CD4^+^ T cells secrete IFNγ and IL-10. CD4^+^ T cells from donors CMV300 and CMV305 specific to gB and UL138 were expanded *in vitro*, LUNA specific cells were also expanded from donor CMV300. Antigen specific CD4^+^ T cells were then incubated with autologous LCL in the presence or absence of peptide and supernatants removed and assayed for 11 cytokines by multi-analyte cytokine assays (A). Supernatants from the same experiment were also used in TGFβ ELISA (B). gB, UL138 and LUNA specific CD4^+^ T cells were then incubated with autologous irradiated PBMC in the presence and absence of peptide in ELISPOT assays detecting IFNγ (C) and IL-10 (D) including the mitogen PHA as a positive control. Error bars are standard error of the mean (n = 5).

CD4^+^ T cells specific to the lytic protein gB induced high levels of the classic T_h1_ type cytokines IFNγ, TNFα and IL2 as expected (Figure 6A) [34], [35]. Both donor CMV300 and CMV 305 responded to the same UL138 peptide (LNVGLPIIGVMLVLI). Interestingly, stimulation of UL138 specific CD4^+^ T cell lines (from donors CMV 300 and CMV305) resulted in a cytokine secretion profile with increased heterogeneity when compared to that observed from gB specific T cells. Specifically we detected the secretion of IFNγ, TNFα, IL-2, IFNβ, IL-6, IL-8 and low levels of IL-4 and IL-5 (Figure 6A). Interestingly, UL138 specific CD4^+^ T cells also produced high levels of the immunomodulatory cytokine cIL-10 in both these donors – an event not seen in response to gB peptide stimulation. Similarly, stimulation of donor CMV 300s' LUNA specific CD4^+^ T cells also produced a heterogeneous range of cytokines including, pertinently, cIL-10 (Figure 6A).

The detection of cIL-10 producing T cells that appeared restricted to the recognition of latently expressed HCMV antigens was intriguing. CD4^+^ T cell production of cIL-10 has been described in a subset of helper T cells associated with immune regulatory functions described as T regulatory cells (T_reg_). T_reg_ are potent modulators of immune responses and exert their effects at least partly via the production of immunomodulatory cytokines, TGFβ and cIL-10. Consistent with this phenotype, both UL138 and LUNA specific CD4^+^ T cells (from donor CMV300) produced TGFβ upon peptide stimulation, while gB specific CD4^+^ T cells from the same donor did not (Figure 6B). A parallel analysis for IFNy and cIL-10 on the T cell lines was performed to assess the frequency of IFNγ and cIL-10 producing cells in the line. As previously, gB, UL138 and LUNA specific CD4^+^ T cells all produced IFNγ upon antigenic stimulation (Figure 6C). Furthermore, UL138 and LUNA, but not gB-specific, CD4^+^ T cells were again shown to secrete cIL-10 (Figure 6D). However, the ELISPOT assays also indicated that the frequency of cIL-10 producing UL138 and LUNA specific T cells was less than the IFNγ producing frequency.

What was not clear from these initial analyses was whether the UL138 specific T cell response we observed was composed of polyfunctional T cells which secrete both pro-inflammatory and immunomodulatory cytokines, or whether the IFNγ and cIL-10 producing T cells were actually separate populations.

To address this, PBMC were stimulated with peptide and then assayed for the production and co-expression of IFNγ and cIL-10 from CD4^+^ T cells by intracellular cytokine staining and flow cytometry. These data show that the stimulation of PBMC from both donors with UL138 peptide resulted in the generation of IFNγ and cIL-10 producing cells, however, the UL138 specific cells were composed of separate populations of CD4^+^ T cells that secreted either IFNγ or cIL-10 and not both (Figure S4A). These data were recapitulated in PBMC from donor CMV300 that exhibited a LUNA specific response composed of separate IFNγ and cIL-10 producing CD4^+^ T cells. A further donor, CMV317, with a known response to both UL138 and LUNA (Table 1, Figure 1 and Figure 2) also showed that both UL138 and LUNA specific CD4^+^ T cells were again composed of separate populations of IFNγ and cIL-10 producing CD4^+^ T cells. Taken together these data show that different subsets of T cells within the CD4^+^ T cell response can be detected and characterised by their cytokine expression profile (summarised in Figure S4B).

Finally, CD4^+^ T cell lines specific to gB and UL138 were also generated from donor CMV305 and, 14 days post *in vitro* expansion, the production of IFNγ and cIL-10 examined in a similar manner. Consistent with our *ex vivo* analyses, the expanded gB specific CD4^+^ T cells were again composed solely of IFNγ producing cells whereas UL138 specific CD4^+^ T cells were again composed of separate populations that produced either IFNγ or cIL-10 (Figure S4C).

### Supernatant from UL138 stimulated CD4^+^ T cells inhibit CD4^+^ T cell proliferation

**Figure 7:**
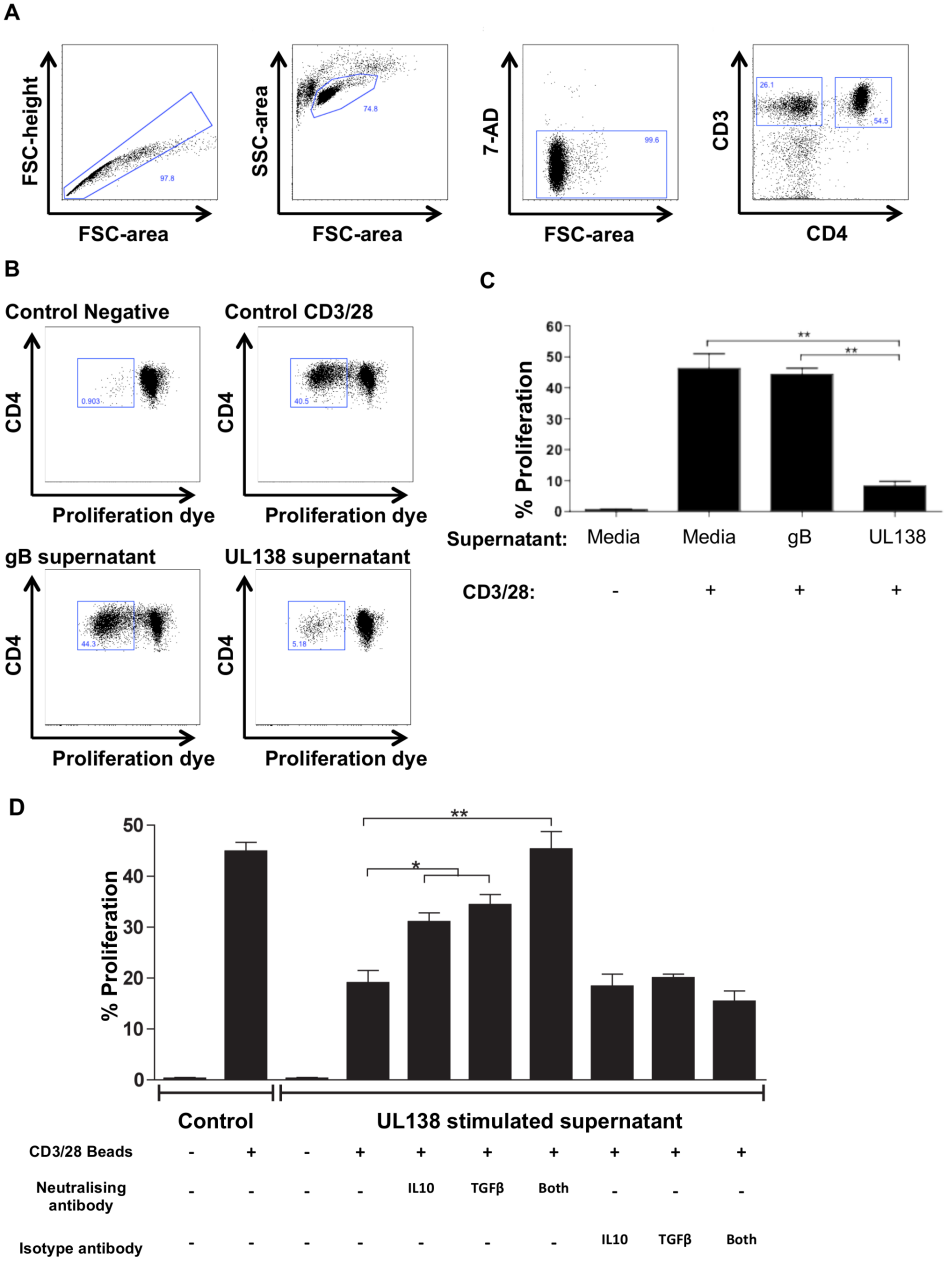
Supernatant from PBMC stimulated with UL138 suppresses CD4^+^ T cell proliferation. PBMC were stimulated with either UL138 or gB peptides for 48(control). PBMC were then stimulated or remained unstimulated (Negative) with anti-CD3/CD28 beads for 5 days and where then stained with anti-CD3 and anti-CD4 antibodies, dead cells excluded by the use of 7-amino-actinomycin D. Cells were gated according to (A) and the proliferation of CD4^+^ T cells was analysed by flow cytometry (B). The same analysis was performed on three individual donors (C). Error bars are standard error of the mean (n = 3). Statistical analysis was performed using the students t test (p<0.01). The same analysis was performed on PBMC from four separate donors and prior to the addition of PBMC supernatants were treated with neutralising anti-IL-10 and/or anti-TGFβ antibodies or isotype control antibodies and proliferation assays performed and analysed as previously (D). Error bars are standard error of the mean (n = 4). Statistical analysis was performed using the students t test (* p<0.05; ** p<0.01).

We reasoned that the observation that UL138 specific CD4^+^ T cells, but not gB specific CD4^+^ T cells, secreted the immunomodulatory cytokines cIL-10 and TGFβ upon stimulation with cognate peptide could potentially result in suppression of the host T cell response. If this was the case then the supernatants from UL138 specific CD4^+^ T cells could impact upon the proliferative response of polyclonally activated CD4^+^ T cells. PBMC from three donors were polyclonally stimulated with anti-CD3/CD28 beads in the presence of supernatant from T cells stimulated for 48 hours with either UL138 or gB specific peptides or media control. Proliferation of CD4^+^ T cells was measured by dye dilution using flow cytometry (Figure 7). Consistent with the prediction that the secretion of cIL-10 and TGF-β was indicative of T_reg_ phenotype we observed that the supernatant from cells stimulated with UL138 (but not gB) peptide, suppressed CD4^+^ T cell proliferation (p<0.01; n = 3) (Figures 7B and C). Supernatant from cells stimulated with UL138 peptide were treated with neutralizing antibodies specific for cIL-10 and TGFβ. The results show that proliferation was partially restored by either neutralizing antibody and in combination proliferation was fully restored to the level of the control (Figure 7D).

### CD4^+^ T cell lines specific for UL138 and LUNA, but not gB include a subset of cells expressing phenotypic markers of T_reg_

The production of the immunomodulatory cytokines cIL-10 and TGFβ, by CD4^+^ T cells has been shown to be associated with the function of a subset of immunosuppressive CD4^+^ T cells termed regulatory T cells (T_reg_) [40]. Indeed, it has been shown previously that the EBV specific CD4^+^ T cell response includes a T_reg_ component specific to a viral gene product expressed during EBV latency [37]. Furthermore, it has also been shown that HCMV can induce the expansion of virus specific CD4^+^ T cells that express phenotypic markers associated with T_reg_ [41].

**Figure 8:**
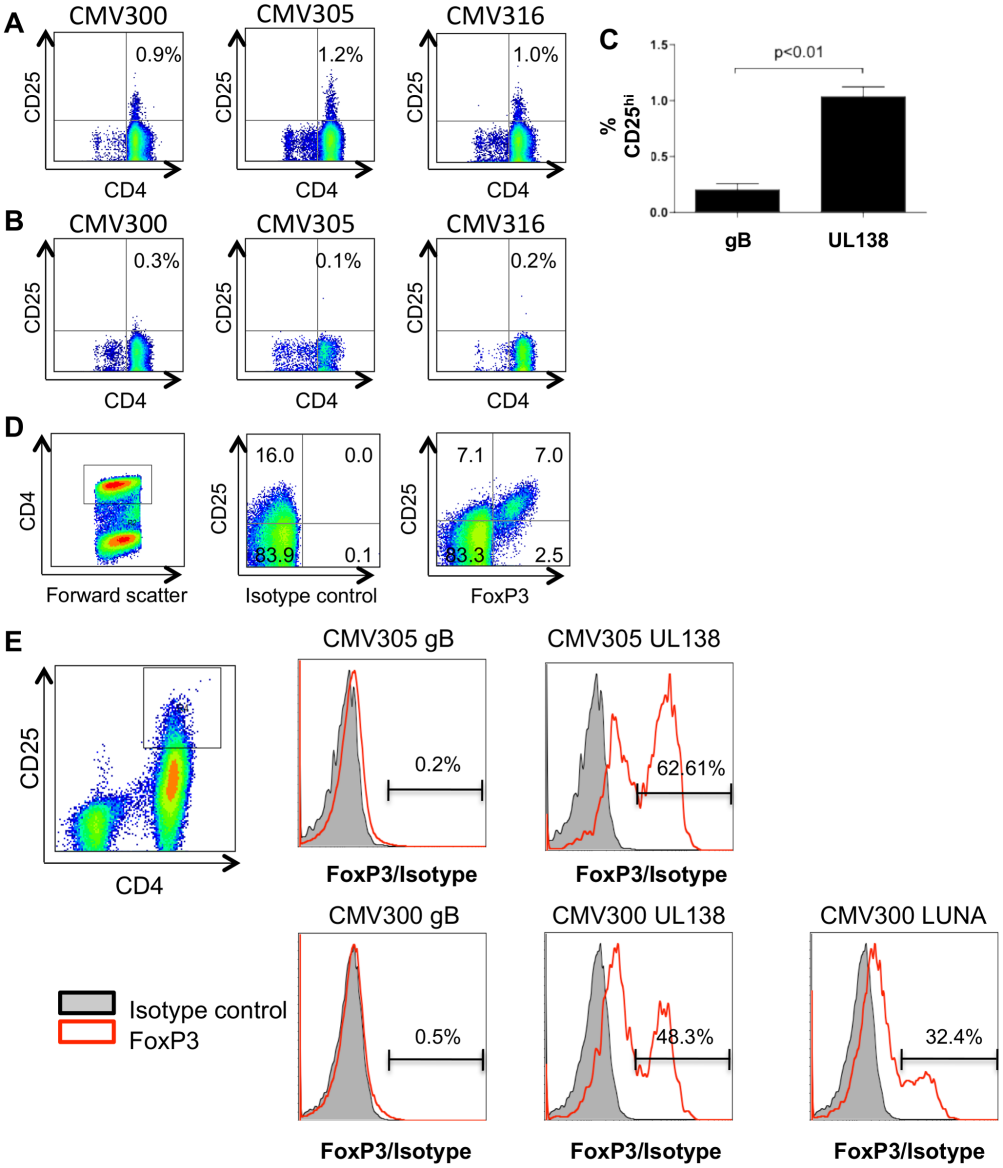
A subset of UL138 specific CD4+ T cells express phenotypic hallmarks of T_reg_ cells. CD4+ T cells specific to UL138 (A) or gB (B) from 3 donors were expanded *in vitro* for 14 days and then stained for stable surface expression of CD25 (C). Numbers in the top right quadrant represent percentage of CD4^+^ CD25^hi^ cells (A and B). Error bars represent standard error of the mean (n = 3); Statistical analysis was performed using the students t test (C). PBMC were stained directly *ex vivo* for surface expression of CD25 and intracellular expression of FoxP3 or using an isotype control (D). Quadrant numbers represent percentage of CD4^+^ cells. In vitro expanded cells from two donors, specific to UL138 and gB (CMV305) or UL138, LUNA and gB (CMV300) were stained for expression of CD4, CD25 and FoxP3 (E). CD4+ CD25hi cells were analysed for expression of FoxP3.

Thus our data so far was highly suggestive that the detection of latent antigens was concomitant with the development of subset of T cells with T_reg_ phenotype. To address this definitively we next examined whether the expanded CD4^+^ T cells specific to UL138, LUNA or gB were populated, in part, with phenotypically defined T_reg_ cells – based on CD4^+^ CD25^hi^ FoxP3^+^ expression [42]–[46]. CD4^+^ T cell lines specific to UL138 (Figure 8A) and gB (Figure 8B) were generated and on day 14 of the expansion protocol assessed for stable expression of CD25. The data clearly showed that UL138 but not gB specific CD4^+^ T cells expressed CD25 after 14 days *in vitro* culture (p<0.01, n = 3) (Figure 8C). We further characterised these cells by determining FoxP3 expression as an indicator of T_reg_ cells. Having established the staining conditions to identify CD4^+^CD25^hi^FoxP3^+^ T cells using whole PBMC (Figure 8D) the CD25, FoxP3 phenotype of CD4^+^ T cells specific to gB, LUNA and UL138, or gB and UL138 from donors CMV300 or CMV305, respectively, were determined (Figure 8E). The results clearly show that CD4^+^CD25^hi^ cells expressing FoxP3, were detected in *in vitro* T cell cultures of UL138 and LUNA specific T cells, but not those specific for gB, in both donors tested. These data showed that a subset of the cells specific for UL138 and LUNA, but not gB, present in the expanded T cell cultures, expressed phenotypic markers consistent with T_reg_ cells.

### Cytokine profile of UL138 and LUNA specific CD4^+^ T cells stimulated directly *ex vivo*

Although unlikely, it was necessary to exclude the possibility that the culture conditions to produce UL138 and LUNA-specific T cell lines induced or favoured cIL-10 producing T cells *in vitro*. Thus, we assayed for cIL-10 production from T cells isolated directly *ex vivo* without prior *in vitro* expansion. In addition, this nature of this analysis allowed us to assess if cIL-10 production by UL138 and LUNA specific T cells was common in a larger panel of donors. Parallel ELISPOT assays detecting IFNγ, cIL-10, IL-4 and IL-17 were thus performed on 13 HCMV seropositive donors using peptides derived from both latent and lytic antigens. The ELISPOT assays for each cytokine (IFNγ, cIL-10, IL-4 and IL-17) were enumerated and the cytokine frequencies were used to determine the percentage of each individual cytokine to the total antigen specific response.

**Figure 9:**
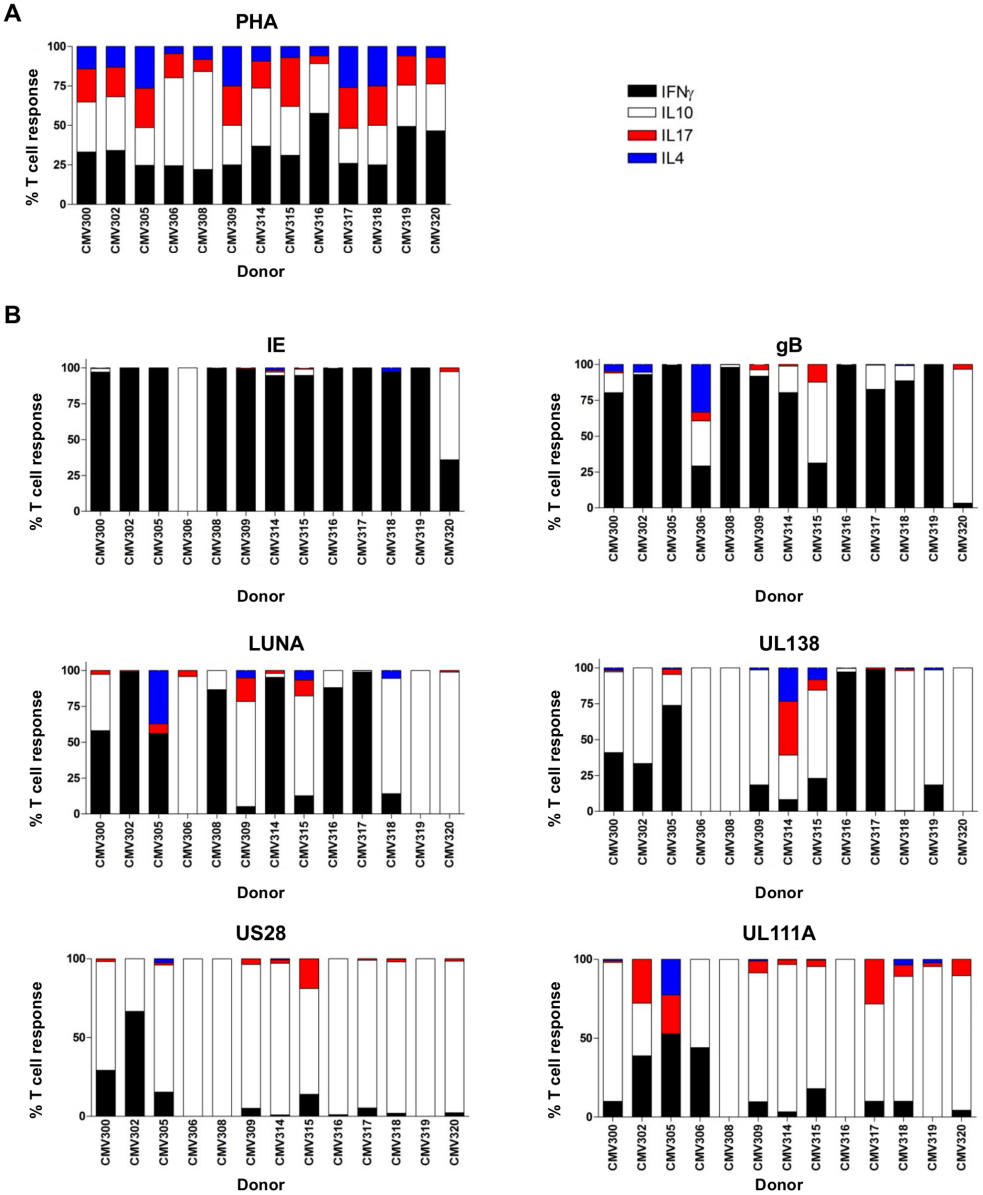
UL138, LUNA, US28 and UL111A specific cells secrete IL-10 and IFNγ directly *ex vivo*. PBMC from 13 HCMV seropositive donors were prepared and used in parallel ELISPOT assays detecting IFNγ (black), IL-10 (white), IL-4 (blue) and IL-17 (red). PBMC were stimulated with PHA (A) or peptide pools spanning HCMV open reading frames IE, gB, UL138, LUNA, US28 or UL111A (B). Post incubation spot forming units (SFU) were enumerated for each cytokine response and the proportion of the total T cell response calculated as a percentage. All samples performed in triplicate, data shown is the mean (Error bars not shown).

Having first confirmed that we could detect all four cytokines in the PBMC from all 13 donors following stimulation with PHA (Figure 9A) we next assayed the effect of specific peptides on cytokine production. As we have demonstrated repeatedly, stimulation with gB, IE, UL138 or LUNA elicited IFNγ responses. IE stimulation was dominated by IFNγ responses while gB elicited predominantly IFNγ however, we note that we did detect a few donors having a small cIL-10 response and 2 donors having a more substantial cIL-10 responding T cell population. In contrast, the cytokine responses to both LUNA and UL138 were much more heterogeneous with most donors producing both IFNγ and cIL-10 and some donors having a predominantly cIL-10 response (Figure 9B). Absolute values (SFU/10^6^ cells) for the total T cell response (IFNγ, cIL-10, IL-4 and IL-17) to each antigen are also shown (Figure S5).

### T cells specific for two additional latency-associated gene products, UL111A and US28, also include CD4^+^ T cells that express both IFNγ and cIL-10

Finally we expanded these analyses to test whether other viral gene products expressed during latency were responsible for a similar T cell phenotype observed with UL138 and LUNA – namely, UL111A (an HCMV homologue of cIL-10) and US28 (an HCMV chemokine receptor homologue) [27]–[32], [47]–[51]. We stimulated the same 13 donors with overlapping ORF peptide pools to UL111A and US28, and measured IFNγ, cIL-10, IL-4 and IL-17 cytokine production in separate ELISPOT assays (Figure 9B). Absolute values (SFU/10^6^ cells) for the total T cell response (IFNγ, cIL-10, IL-4 and IL-17) to each antigen are also shown (Figure S5). These results clearly show that donors did have both UL111A and US28 specific T cells and, importantly, while IFNγ responses could be measured the dominant cytokine to these antigens was cIL-10. Furthermore, we note that some donors also had small IL-4 or IL-17 cytokine responses. Taken together these data clearly show that there is a circulating population of CD4^+^ T cells detectable directly *ex vivo* that recognise latently expressed HCMV antigens and which have a phenotype consistent with the production of the immunomodulatory cytokine cIL-10.

## Discussion

The results we present here represent the first comprehensive analysis of T cell responses to those viral proteins associated with latent HCMV infection. In general, and in contrast to antigens solely associated with virus lytic infection (such as IE, gB and pp65), our results show that healthy seropositive donors have robust T cell responses to all the latency-associated antigens we analysed, which are dominated by CD4^+^ T cells. As expected, these CD4^+^ T cells recognise cells lytically infected with HCMV but, importantly, also recognise latently infected monocytes.

There have been few other analyses of T cell responses to HCMV encoded ORFs associated with latent infection. In a total proteome screen for HCMV-specific T cell responses undertaken by Sylwester *et al* (2005) only one donor out of 33 was identified as having a UL138-specific CD4^+^ and CD8^+^ T cell response whereas LUNA was not included in their analysis [21]. Similarly, an independent analysis of UL138 identified UL138-specific CD8^+^ T cell responses, but only in individuals who expressed the HLA-B3501 haplotype [33]. Our study also included four HCMV seropositive donors who expressed HLA-B3501, but none of these individuals had detectable CD8^+^ T cell responses to UL138 in our hands. These differences could be due to the methods used to detect antigen specific T cells - our studies used ELISPOT assays to screen directly *ex vivo*, in contrast to an *in vitro* antigen stimulation to induce T cell expansion and pre-enrichment prior to detection of IFNγ used in the previous study [33]. Also, in contrast to Tey *et al* (2010), who reported an absence of CD4^+^ T cell responses to either UL138 or LUNA, we observed robust CD4^+^ T cell responses to these antigens in healthy donors. Again, it is likely that experimental differences between the studies, such as the size of the individual peptides, the use of the ELISPOT assays versus intracellular cytokine detection and the duration of the assay (48 hours v 6 hours restimulation) may account for these discrepancies.

Consistent with the knowledge that UL138 and LUNA are also expressed during lytic infection, our data clearly showed that CD4^+^ T cells specific to these viral proteins were able to recognize lytically infected moDCs. Furthermore, UL138 specific CD4^+^ T cells expanded *in vitro* were also able to mediate cytotoxicity against peptide-loaded autologous target cells. This recognition of lytically infected DCs, and concomitant secretion of IFNγ, occurred despite the expression of those viral genes associated with lytic infection that are known to modulate immune responses [3], [52]–[55]. However, this is not inconsistent with numerous studies which have shown potent anti-viral CD4^+^ T cell responses to other antigens such as gB and IE (expressed only during lytic infection) in HCMV infected cells, despite expression of the known viral immune-evasins [34], [56].

Our observations that CD4^+^ T cells specific for UL138 could also recognise latently infected cells and secrete IFNγ leads to an obvious conundrum: this ability of the host to recognise latently infected cells carries the risk that the latently infected cells should become targets for immune clearance. However, it is already known that HCMV is able to modify the latently infected cell itself in order to reduce T cell recognition and activation. During latent infection expression of viral UL111A (vIL-10) results in down regulation of MHC class II and diminished CD4^+^ T cell recognition [49] and latent infection of CD34^+^ bone marrow progenitor cells induces release of cIL-10 and TGFβ which decreases CD4^+^ T cell IFNγ production and cytotoxicity [14]. We have not been able to determine if LUNA protein expression in latently infected cells can be recognized by LUNA specific T cells and thus this remains an open question.

The results presented here now demonstrate that both UL138 and LUNA-specific CD4^+^ T cells also, themselves, secrete the immunomodulatory cytokine cIL-10, in direct contrast to CD4^+^ T cells specific for gB and IE antigens (which are expressed solely during lytic infection). Interestingly, our analysis of the latency-associated antigens US28 and UL111A also showed a skewing of T cell responses towards CD4^+^ T cells which secreted cIL-10 suggesting that immune evasion during latency is a complex interplay between the microenvironment around the latently infected cell and the properties of the immune cells recruited to it.

In the donors analysed directly *ex vivo*, there was a clear bias towards CD4^+^ T cells which secreted cIL-10 in response to the latency associated HCMV gene products UL138, LUNA, UL111A and US28. Not all donors respond to every latency associated gene product, however, when all four antigens (UL138, LUNA, US28 and UL111a) were taken together for the 13 donors tested two donors made no responses, one donor made an IFNã response and the remaining 10 donors had an cIL-10 response to at least one of the latently expressed antigens. Indeed, if a donor did not make a T cell response to any latently expressed antigen there is no requirement for cIL-10 producing latent antigen specific T cells. Regarding the single donor we found that makes an IFNã response but no associated cIL-10 response to latent antigens it is possible that there are other latent antigens that have yet to be recognised. We have recently published that UL144 is also expressed during latency [57] and it is possible that this donor makes cIL-10 to this antigen. Alternatively, it is possible that some donors do make antiviral responses to latent antigens that are not balanced with a cIL-10 T cell response and that these individuals may turn out to have lower latent viral loads than individuals with higher frequency cIL-10 responses.

Interestingly, EBV also induces high frequencies of CD4^+^ T cells specific to latent antigens; targeting EBNA1 which suppress the proliferation and cytokine production of both CD4^+^ and CD8^+^ T cells [58] and LMP1 that also secrete cIL-10 [37]–[39]. cIL-10 secreting CD4^+^ T cells are known to perform an immunomodulatory role in the immune response, often functioning to restrict immune activation [59]–[61] and are a classical signature of T_reg_ cells [40], [59]. It was interesting to note that the UL138 and LUNA T cell lines expanded *in vitro* more slowly than the gB specific T cell lines (data not shown). We were also able to show that the suppression of polyclonally activated CD4^+^ T cell proliferation was due to cIL-10 and TGFβ secreted in the supernatant from UL138 specific T cells. In MCMV latency, cIL-10 producing CD4^+^ T cells have been isolated from salivary glands. Furthermore, in cIL-10 knockout mice (or after IL-10R blockade) the latent MCMV load is reduced [62] with a concomitant increase in memory MCMV-specific T cell frequency being observed. These observations are consistent with the view that cytomegalovirus may induce cIL-10 producing CD4^+^ T cells to prevent latently infected cells from being recognised by the immune system [63].

In contrast to EBV, it was not known whether viral gene products expressed during HCMV latency generate T_reg_ cells. Lytic HCMV (IE and pp65) antigen specific T_reg_ cells have been described, particularly enriched in kidney transplantation patients that had recurring HCMV reactivation events. The authors suggested that frequent episodes of antigen stimulation might drive the T_reg_ phenotype and they further demonstrate that the antigen specific T_reg_ cells had the same TCR as effector T cells, suggesting a common lineage [41]. IE and pp65 specific T_reg_ cells were also isolated from normal healthy donors but at lower frequencies than in recurrent patient groups. In this study we examined IE and gB specific CD4^+^ T cells and in some healthy individuals we were able to identify cIL-10 producing cells in addition to predominant IFNγ secreting cells, which is in agreement with the Schwele observations. However, key to this study, is the detection of cIL-10 production by UL138 and LUNA specific CD4^+^ T cells in a larger number of donors than was seen with IE and gB specific CD4^+^ T cells. Indeed, the number of donors that had cIL-10 secreting T cells specific for US28 and UL111A was striking and within individual donors was often dominant over IFNγ producing T cells of the same specificity.

It is unclear why CD4^+^ T cell responses to HCMV lytic antigens appear to be dominated by IFNγ producing cells, while CD4^+^ T cells, which recognise antigens, expressed during latent infection predominantly secrete cIL-10. Many factors are likely to impact upon the type of CD4^+^ T cell response generated to a particular antigen, and this is known to include the cytokines present in the microenvironment during T cell activation. During latent infection, viral gene expression in CD34^+^ cells is highly restricted and associated with secretion of immunomodulatory cytokines cIL-10 and TGFβ [14]. This immunosuppressive microenvironment may also have an impact on the generation of CD4^+^ T cells activated during latent phases of infection. Specifically, the CD34^+^ mediated secretion of cIL-10 and TGFβ, may result in the generation of cIL-10 producing, immunomodulatory CD4^+^ T cells specific to latent antigens but not those expressed solely during lytic infection [64]–[67]. It is highly plausible that these effects act in concert with the known functions of latency-associated UL111A (vIL-10) which has been shown to promote MHC class II down-regulation and inhibit CD4^+^ T cell activation [29]–[31], [49] as well as restrict the ability of latently infected myeloid cells to differentiate. This ability to modulate the ability of the infected cell to function as a professional antigen presenting cell as has been seen during HSV infection of plasmacytoid DCs [68], [69].

It has been shown that that some HCMV seropositive donors generate cIL-10 secreting T cells to lytic HCMV antigens (such as pp65) [41], in agreement with these observations we have also seen cIL-10 producing T cells specific for gB and IE in a small number of donors tested. Schwele et al clearly demonstrated that these cIL-10 producing T cells were generated at a higher frequency in reactivating transplant individuals (speculated to be due to repeated antigenic stimulations) and, as our cohort were normal healthy donors, this probably accounted for the lower frequency of detection in our analysis. It might be expected that in older HCMV seropositive donors, who have carried the virus for many years, that you would see the generation of cIL-10 producing CD4^+^ T cells to lytic antigens and maybe an increase in frequency to latent antigens. Our data highlights the consistent generation of cIL-10 producing CD4^+^ T cells, in most normal healthy donors, to antigens expressed in latency and thus the possibility that this is important in preventing the immune clearance of latently infected cells.

We believe that there are potential clinical implication from these findings, in the case of bone marrow transplantation (D+/R−) if it were possible to eliminate or drastically reduce the latent viral load prior to transplantation this could either prevent reactivation or substantially reduce reactivation loads. We speculate that since the T cell response to the latent antigens is composed of both anti-viral (T_h1_) and immunesuppressive activities the neutralization of cIL-10/TGFβ may allow Th1 type latent specific T cells to recognize and results in the elimination of latent CD34^+^ cells.

In conclusion, based on this and other studies we suggest that a number of direct and indirect immune suppressive mechanisms may act together to help maintain sites of HCMV latency: Virally encoded UL111A (vIL-10) expressed during latency down regulates MHC class II expression on APCs restricting T cell recognition of latently infected cells [49]; latent infection of CD34^+^ cells results in increases in cIL-10 and TGFβ in the cell secretome, which act to suppress antiviral immune responses in the microenvironment of latently infected cells [14] and now we show that CD4^+^ T cells specific for viral latency-associated gene products, themselves secrete cIL-10 which helps suppress antiviral effector functions. This biasing of the immune response by latency-associated antigens, to elicit CD4^+^ T cells that secrete cIL-10, may assist in the maintenance of the latent reservoir and lifelong carriage of HCMV *in vivo*.

## Materials and Methods

### Ethics statement

Ethical permission for this project was granted by the Cambridgeshire 2 Research Ethics Committee (REC reference 97/092). Informed written consent was obtained from all of the volunteers included in this study prior to providing blood samples.

### Identification of donor HCMV serostatus

HCMV serostatus of 23 healthy volunteers was determined using a commercial HCMV specific IgG ELISA kit (Captia, Trinity biotech, Ireland). The assay was performed according to manufacturer's instructions.

### Human leukocyte antigen typing of donors

Human leukocyte antigen typing was performed for donors regardless of HCMV serostatus. All MHC class I alleles (HLA-A, HLA-B, HLA-C) and HLA-DR and HLA-DQ MHC class II alleles were typed by molecular methods by Helen Stevens (Cambridge Institute for Medical Research, UK) (Table S1).

### Peptide libraries

Sequences for viral proteins from the clinical HCMV strain Merlin were used and peptides constructed as sequential 15 amino acid peptides with 10 amino acid overlap, spanning UL138 (Table S2A), LUNA (), US28 (Table S2C) and UL111A (Table S2D) gB, IE and pp65 from Proimmune (UK). Peptides were reconstituted and stored according to manufacturer's instructions to give a storage concentration of 40 mg/ml. Individual peptides were further diluted in RPMI 1640 (PAA laboratories, Austria) to create a stock of 1 mg/ml and a working concentration of each peptide of 40 µg/ml and stored at −80°C. Peptide pools were made for screening purposes from the single peptides, and were constructed to give 2 µg/ml of each individual peptide. These peptide pools were then stored in 100 µl aliquots at −80°C.

### Preparation of peripheral blood mononuclear cells (PBMC)

Venous blood was collected in heparin sodium (100 IU/ml), diluted 1∶2 with RPMI-1640 containing no serum (PAA laboratories, Austria) supplemented with 100,000 IU/ml penicillin, 100 mg/ml streptomycin, and 2 mmol/ml _L-_glutamine (RPMI-wash). Peripheral blood mononuclear cells (PBMC) were isolated by Lymphoprep (Axis-Shield, Norway) centrifuged at 800 g for 15 minutes. Autologous serum was removed from the interface and incubated at 60°C for 30 minutes in a water bath to inactivate complement.

### Transformation of donor derived lymphoblastic B cell lines

LCL lines were established according to published protocols [70].

### Detection of cytokine production by ELISPOT assays

ELISPOT plates were prepared, coated and blocked according to manufacturer's instruction (EBioscience). PBMC directly *ex vivo*, previously frozen, or depleted of either CD4^+^ or CD8^+^ T cells by magnetic activated cell sorting (MACS), were plated 3.0×10^5^ cells in 100 µl RPMI-10 per well (of a 96 well Multiscreen IP sterile plate (Millipore, UK)). Plates were incubated for 48 hours at 37°C 5% CO_2_, and developed according to manufacturer's instruction. Plates were read using an ELISPOT plate scanner (ELISPOT Reader System, AID) and spots enumerated using ImageJ (National Institutes of Health).

### Depletion of CD4^+^ or CD8^+^ T cells from PBMC

PBMC were depleted of either CD4^+^ or CD8^+^ T cells by MACS using either anti-CD4^+^ or anti-CD8^+^ direct beads (Miltenyi, U.K.), according to manufacturer's instructions and separated on LS columns (Miltenyi, U.K.). Efficiency of depletion was determined by staining cells with either anti-CD4 or anti-CD8 antibodies and analysed by flow cytometry. Depletions performed in this manner resulted in 0.1–0.8% CD4^+^ cells and 0.3–0.8% CD8^+^ cells, respectively.

### In-vitro expansion of antigen specific CD4^+^ T cells

CD4^+^ T cells were purified by MACS using anti-CD4 direct beads (Miltenyi, UK) and separated on LS columns (Miltneyi, UK) according to manufacturer's instructions which resulted in CD3^+^ CD4^+^ mean cells purities of 98.1% (range 95.6–99.8%) as determined by flow cytometry. PBMC (5.0×10^6^) were incubated with 100 µl of the peptide of interest (2 µg/ml) for two hours at 37°C before irradiation using a sealed source irradiator for 30 minutes. These cells were then washed in PBS, resuspended in RPMI-1640 supplemented with 10% autologous donor serum, 100,000 IU/ml penicillin, 100 mg/ml streptomycin, and 2 mmol/ml _L-_glutamine. PBMC were then transferred 5.0×10^5^ cells per well of a 96 well round bottomed microtitre plate. MACS purified CD4^+^ T cells were then added 1.0×10^4^ cells per well and incubated at 37°C for 14 days. RPMI-1640 supplemented with 10% autologous donor serum, 100,000 IU/ml penicillin, 100 mg/ml streptomycin, and 2 mmol/ml _L-_glutamine and 15 IU/ml recombinant IL-2 (National Institute of Biological Standards and Control, U.K) was added 50 µl per well on days 2, 8 and 12 of culture.

### Detection and quantification of cytokine production by multi-cytokine assay

The production of cytokines by peptide specific T cell lines was determined using Flowcytomix T_h1_/T_h2_ 11plex kit, or using simplex kits for specific cytokines (Bendermed systems, Netherlands) according to manufacturer's instructions. Samples were analysed using a BD FACSort and data analysed using Flowcytomix pro software (Bendermed systems, Netherlands).

### Detection of TGFβ production by ELISA

Supernatants from peptide stimulated CD4^+^ T cells were assayed for the presence of TGFβ by ELISA, according to manufacturer's instructions (R&D systems, U.K.).

### Intracellular detection of cytokine production by flow cytometry

PBMC were washed in PBS and 1.5×10^6^ cells were resuspended in 500 ul RPMI-1640 supplemented with 10% autologous donor serum, 100,000 IU/ml penicillin, 100 mg/ml streptomycin, and 2 mmol/ml _L-_glutamine, in polypropylene FACS tubes (B.D., U.K.). Cells were then incubated with 100 µl (40 µg/ml) of the peptide of interest and incubated for 16 hours at 37°C +5% CO_2_. Post incubation Brefeldin A and Monensin (Biolegend, U.K.) were added to the cultures according to the manufacturers instructions, and the tubes incubated for a further 4 hours. Post incubation cells were stained with a LIVE/DEAD fixable dead cell stain kit (Invitrogen, U.K.), according to manufacturers instructions. Cells were then surface stained with anti-CD3 PE-Cy7 (B.D., U.K.) and anti-CD4 PerCP Cy5.5 (B.D., U.K.) monoclonal antibodies according to manufacturers instructions. Intracellular cytokines were fixed and permeablised using the FoxP3/Transcription factor staining buffer set (EBioscience, U.K.). Intracellular cytokines were stained using anti-IFNγ alexafluor 488 (Biolegend, U.K.) and anti-IL10 PE (Biolegend, U.K.) and acquired using the FACS Canto II (B.D., U.K.) and analysed using FlowJo software (Treestar, U.S.A).

### Flow cytometric detection of T_reg_ cells

Expanded T cell lines or PBMC were assayed for the presence of T_reg_ using the Human Regulatory T cell staining kit (EBioscience, U.K.), according to manufacturer's instructions.

### T cell cytotoxicity assays

Cultured peptide specific T cell lines were used in a standard Cr^51^-release assay against both HLA matched and mis-matched B cell lines as target cells as previously described [70].

### T cell suppression assays

PBMC were labelled with cell trace violet proliferation kit for flow cytometry (Life technologies, U.K.), according to manufacturer's instructions. Cells were then resuspended in RPMI-1640 supplemented with 10% autologous donor serum, 100,000 IU/ml penicillin, 100 mg/ml streptomycin, and 2 mmol/ml _L-_glutamine. Alternatively, PBMC were resuspended in supernatant from PBMC cultures stimulated for 48 hours with gB or UL138 peptides, and plated 1.0×10^5^ per well of a round bottom 96 well plate and incubated for 1 hour at 37°C +5% CO_2_. For neutralisation assays, supernatants were treated with neutralising antibodies for cIL-10, TGFβ or both, or with the relevant isotype control antibodies at a final concentration of 20 ng/ml for one hour prior to the addition of the violet labelled PBMC. Post incubation cells were stimulated with Dynabeads® Human T-Activator CD3/CD28 (Life technologies, U.K.) at a bead to cell ratio of 1∶200. Alternatively cells remained unstimulated and all wells were adjusted to a total volume of 200 µl and incubated for 5 days at 37°C +5% CO_2_. Post incubated cells were harvested and washed in PBS prior to staining with anti-CD3 PE-Cy7 (B.D., U.K.) and anti-CD4 FITC (B.D., U.K.) antibodies. Cells were then stained with 7-amino- Actinomycin D (Calbiochem, U.K.). Cells were then washed in PBS and resuspended in FACS buffer and acquired on the FACS Canto II and analysed FlowJo software.

### Generation of lytically infected dendritic cells

Monocytes were isolated from PBMC using anti-CD14 MACs beads according to the manufactures instructions (Miltneyi, UK). The purity of isolated monocytes was determined by flowcytometric detection of CD14^+^ cells, resulting in mean CD14+ populations of 98.1% (range 97.4–98.9%, n = 5). Purified monocytes were adhered to tissue culture plates overnight in X-vivo15, medium was then changed to X-vivo15 supplemented with 2.5 mM _L_-glutamine, 500 IU/ml IL-4 (Peprotech, UK) and 1000 IU/ml granulocte-macrophage colony stimulating factor (GM-CSF) (Peprotech, UK) and incubated for a further 3 days at 37°C + 5% CO_2_. Post incubation cells were washed in PBS and fresh X-vivo15 supplemented with 2.5 mM _L_-glutamine, 500 IU/ml IL-4 (Peprotech, UK) and 1000 IU/ml GM-CSF (Peprotech, UK) was added for a further 3 days. Post incubation, the cells were washed in PBS and matured by the addition of 1 ml X-vivo15 per well supplemented with 2.5 mM _L_-glutamine and 50 ng/ml LPS for 48 hours. Cell surface phenotype of monocytes and the iDC and mDC cells derived from them was determined by flow cytometry using anti-CD14, CD80, CD86 and HLA-DR, as expected iDC and mDC lost CD14 and gained CD83 expression, MHC Class II, CD80 and CD86 were all upregulated (Figure S1). *In vitro* differentiated monocyte derived dendritic cells were infected at MOI 5 for 3 hours at 37°C + 5% CO_2_ in X-vivo15 supplemented with 2.5 mM _L_-glutamine. Post incubation supernatant was removed and 1 ml X-vivo15 supplemented with 2.5 mM _L_-glutamine added per well and further incubated at 37°C + 5% CO_2_. Media was changed every 3 days post infection and cells used as antigen presenting cells 5 days post infection in the presence or absence of cognate peptide. Infection was confirmed by immunofluorescence and RT-PCR (see below).

### Generation of latently infected monocytes

Monocytes were isolated from PBMC using anti-CD14 MACs beads according to the manufactures instructions (Miltneyi, UK) and adhered in tissue culture plates overnight in X-vivo15 supplemented with 2.5 mM _L_-glutamine overnight at 37°C + 5% CO_2_. Post incubation cells were washed in PBS and infected with TB40e UL32GFP at an MOI of 5 for 3 hours at 37°C + 5% CO_2_. Post incubation cells were washed in PBS and 1 ml fresh X-vivo15 supplemented with 2.5 mM _L_-glutamine added and incubated 37°C + 5% CO_2_ for 10 days. Medium was changed every 3 days post infection and cells used as antigen presenting cells 10 days post infection in the presence or absence of peptide. Establishment of latency was confirmed at day ten by RT-PCR.

### Immunofluorescence IE staining

Cells were fixed in 4% PFA in PBS for 10 minutes at room temperature, washed twice in PBS prior to the addition of 0.1% Triton-X in PBS for ten minutes at room temperature. 300 µl mouse anti-IE antibody (1∶1000; Millipore, UK) per well for 1 hour at room temperature, followed by PBS washes and then stained with anti-mouse Alexafluor 594 (1∶1000; Invitrogen, UK) and DAPI (1∶100; Invitrogen, UK) in 300 µl PBS for 1 hour at room temperature in the dark. After washing cells were analysed immediately by fluorescent microscopy.

### RT-PCR of viral transcripts

RNA was extracted from *in vitro* cell infections using a previously published method [71]. Briefly, adherent cells were washed in chilled PBS and 1 ml TRIZOL (Invitrogen, UK) added per well (for a maximum of 1×10^6^ cells), adherent cells were removed using a cell scraper. RNA samples were DNase treated using the RQ1 RNase free DNase kit (Promega, UK) according to manufacturer's instructions. Samples were then reversed transcribed using the Reverse transcription system (Promega, UK) according to manufacturer's instructions. PCR was performed to amplify a range of viral transcripts associated with either lytic or latent infection, or cellular genes: GAPDH forward GAGTCAACGGATTTGGTCGT and GAPDH reverse TTGATTTTGGAGGGATCTCG [72]; IE forward GGACCCTGTAATCCTGACG and IE reverse ATCTTTCTCGGGGTTCTCGT [73]; UL138 forward TGCGCATGTTTCTGAGCTC and UL138 reverse ACGGGTTTCACAGATCGAC [28]; LUNA forward ATGACCTCTCCTCCACACC and LUNA reverse GGAAAAACACGCGCGGGGGA [27] (all primers obtained from Sigma-Adlrich, UK). 45 cycle PCR Biomix red (Bioline, UK), was performed using: 95°C 1 minute, 55°C 40 seconds, 72°C 1 minute.

## Supporting Information

Figure S1 **Differentiation and phenotype of monocytes and monocyte derived dendritic cells.** Monocytes were prepared from PBMC by CD14 selection and cultured *in vitro*. Alternatively, monocytes were differentiated using IL-4 and GM-CSF to immature dendritic cells (iDC) and then activated with lipopolysaccharide to mature dendritic cells (mDC). All three cell types were then stained with monoclonal antibodies specific for CD14, CD83, HLA DR, CD86 and CD80 to determine their phenotype by flow cytometry.
(TIF)

Figure S2 **CD4^+^ T cell detection of lytic infection in monocyte derived dendritic cells.** Dendritic cells (A, C and E) or Macrophaages (B, D and F) were prepared from donor CMV305 and mock infected or lytically infected with TB40e for 5 days at MOI 5. Lytic infection was then confirmed by RT-PCR (G). Autologous mock or TB40e infected dendritic cells or monocytes were then co-incubated with *in vitro* expanded antigen specific CD4^+^ T cells specific to gB, IE or UL138 in IFNγ ELISPOT assays in the presence or absence of cognate peptide (A–F). Post incubation IFNγ spot forming units (SFU/10^6^) were enumerated and the back ground level of IFNγ production for each antigen specificity determined from the mock infected no peptide control (Red dotted line).
(TIF)

Figure S3 **UL138 specific CD4^+^ T cells secrete IFNγ in response to latently infected monocytes.** Monocytes were prepared from donor CMV300 and mock infected or latently infected with TB40e for 10 days at MOI 5. Latent infection was then confirmed by RT-PCR (A). Autologous mock or latently infected monocytes were then co-incubated with *in vitro* expanded antigen specific CD4^+^ T cells specific to gB (B) and UL138 (C) in IFNγ ELISPOT assays in the presence or absence of cognate peptide. Post incubation IFNγ spot forming units (SFU/10^6^) were enumerated and the back ground level of IFNγ production for each antigen specificity determined from the mock infected no peptide control (Red dotted line). Error bars are standard error of the mean (n = 5). Statistical analysis were performed using the students t test (* p<0.05;** p<0.01).
(TIF)

Figure S4 **The UL138 specific T cell response is composed of separate populations of IFNγ and cIL-10 producing CD4^+^ T cells.** PBMC from three seropositive donors were stimulated with a range of peptides: CMV300 gB, UL138 and LUNA; CMV305 gB and UL138; CMV317 UL138 and LUNA, and intracellular IFNγ and cIL-10 were detected by flow cytometry gating on the live CD3^+^ CD4^+^ lymphocyte population (A). Quadrant values represent % of the total CD3^+^ CD4^+^ population for the Unstimulated (US) stimulated sample. Values for the US were used to determine background cytokine secretion and subtracted for sample stimulated with peptide. The proportion of the responding population was then plotted for the percentage of the total cytokine positive response for each donor and peptide stimulation: IFNγ+IL10− (White); IFNγ+IL-10+ (Grey) and IFNγ-IL-10+ (Black) (B). UL138 and gB specific CD4+ T cells from donor CMV305 were expanded *in vitro* for 14 days and then stimulated with peptide prior to intracellular detection of IFNγ and cIL-10 by flowcytometric methods and analysis of the live CD3^+^ CD4^+^ lymphocyte population (C). Background cytokine production for each line was determined by an unstimulated control (No peptide). Quadrant values show the percentage of the live CD3^+^ CD4^+^ lymphocyte population for each condition.
(TIF)

Figure S5 **Quantification of the T cell response to IE, gB, UL138, LUNA, US28 and UL111A by multiple cytokine specific ELISPOT assay.** PBMC from 13 seropositive donors were stimulated with overlapping peptide pools spanning the HCMV open reading frames IE, gB, UL138, LUNA, US28 and UL111A in separate ELISPOT assays detecting IFNγ, IL-10, IL-4 and IL-17. Post incubation assays were developed and spot forming units (SFU) for each cytokine enumerated using ImageJ. Background levels of cytokine production from each donor were determined from an unstimulated control, subtracted from the corresponding cytokine specific assay prior to conversion to SFU/106 cells. Finally, values for each individual cytokine were used to calculate a cumulative cytokine response (including all four cytokines). Error bars represent standard error of the mean (n = 3).
(TIF)

Table S1 **Serostatus and HLA type of the cohort.** All donors were serologically type for HCMV IgG by ELISA (+) HCMV seropositive (−) HCMV seronegative. All donors were also HLA typed for HLA-A, B, C and HLA-DR and DQ by molecular methods. (*) Unable to type further.
(DOCX)

Table S2 **Peptide sequences of individual 15 amino acid peptides of UL138 and LUNA.** 32 overlapping 15mer peptides of UL138 (A) and 20 overlapping 15mer peptides of LUNA (B) and (C) 35 overlapping 15mer peptides of UL111A and (D) 69 overlapping 15mer peptides of US28.
(DOCX)
