# Supplementary figures and images for: Human Cytomegalovirus Latency-Associated Proteins Elicit Immune-Suppressive IL-10 Producing CD4+ T Cells
Source: PLoS Pathog. 2013 Oct 10;9(10):e1003635. doi: 10.1371/journal.ppat.1003635 (PMC3795018; doi:10.1371/journal.ppat.1003635)

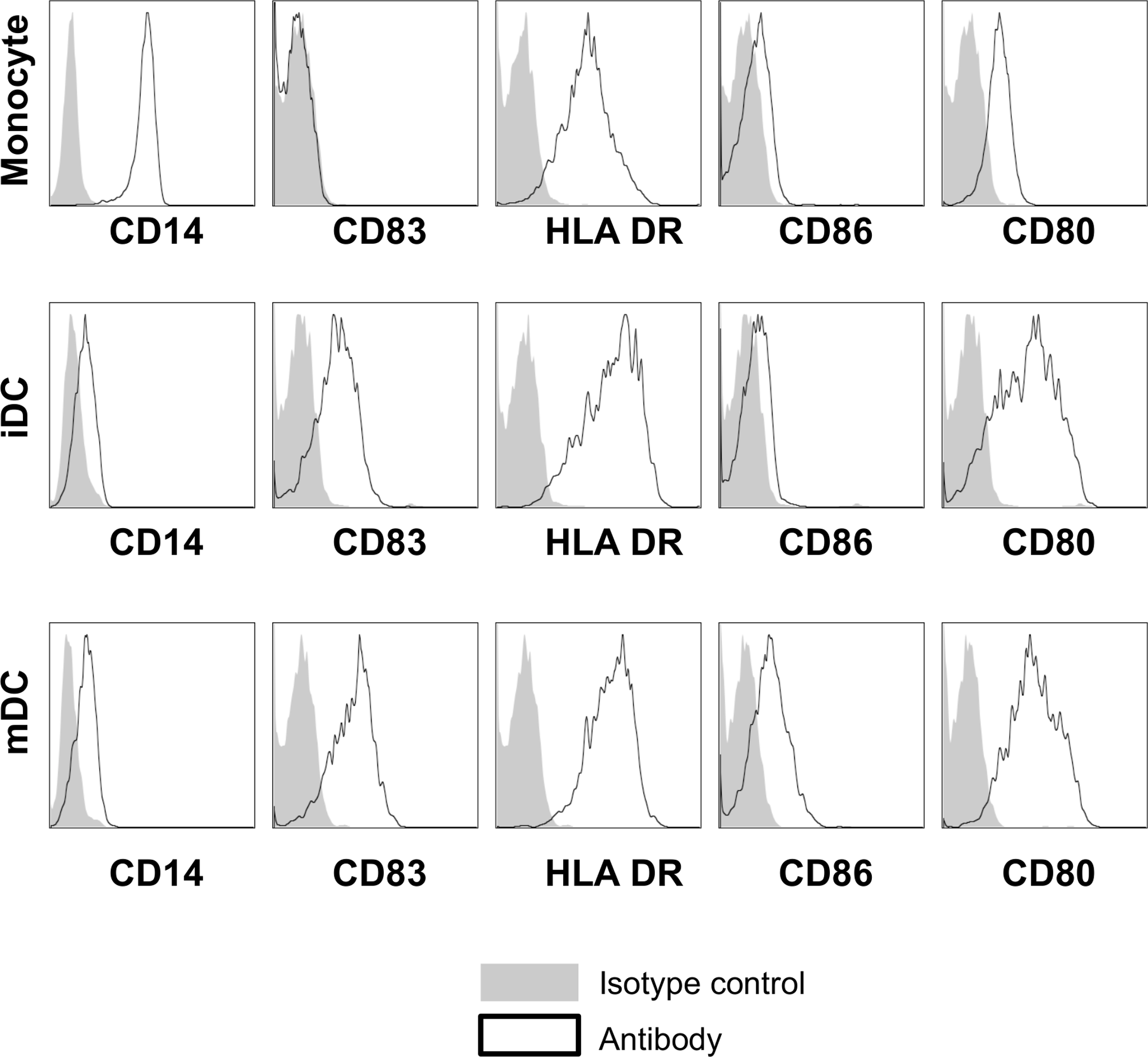

Supplement: Figure S1 — Differentiation and phenotype of monocytes and monocyte derived dendritic cells. Monocytes were prepared from PBMC by CD14 selection and cultured in vitro. Alternatively, monocytes were differentiated using IL-4 and GM-CSF to immature dendritic cells (iDC) and then activated with lipopolysaccharide to mature dendritic cells (mDC). All three cell types were then stained with monoclonal antibodies specific for CD14, CD83, HLA DR, CD86 and CD80 to determine their phenotype by flow cytometry. (TIF) [file ppat.1003635.s001.tif]

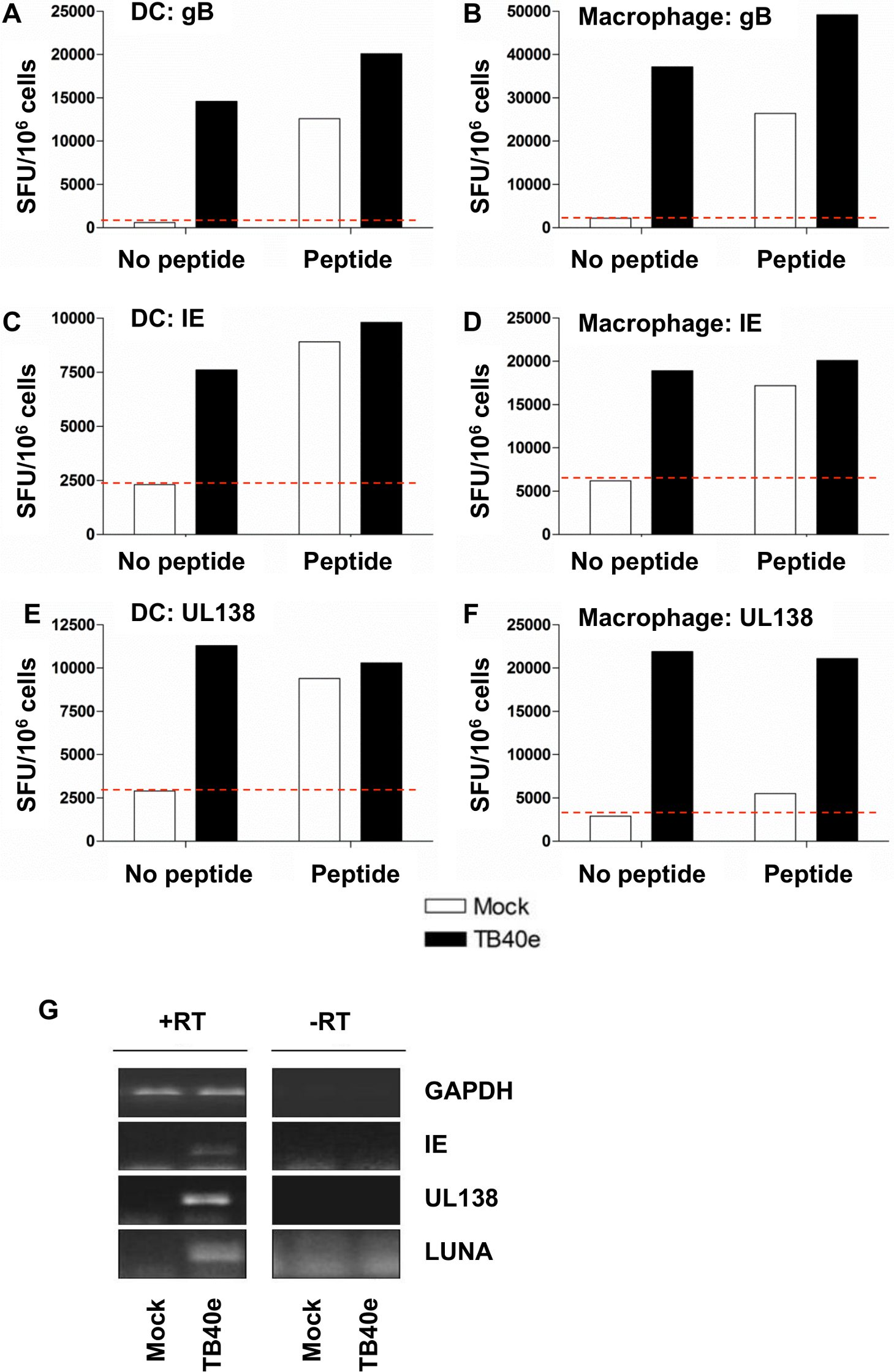

Supplement: Figure S2 — CD4+ T cell detection of lytic infection in monocyte derived dendritic cells. Dendritic cells (A, C and E) or Macrophaages (B, D and F) were prepared from donor CMV305 and mock infected or lytically infected with TB40e for 5 days at MOI 5. Lytic infection was then confirmed by RT-PCR (G). Autologous mock or TB40e infected dendritic cells or monocytes were then co-incubated with in vitro expanded antigen specific CD4+ T cells specific to gB, IE or UL138 in IFNγ ELISPOT assays in the presence or absence of cognate peptide (A–F). Post incubation IFNγ spot forming units (SFU/106) were enumerated and the back ground level of IFNγ production for each antigen specificity determined from the mock infected no peptide control (Red dotted line). (TIF) [file ppat.1003635.s002.tif]

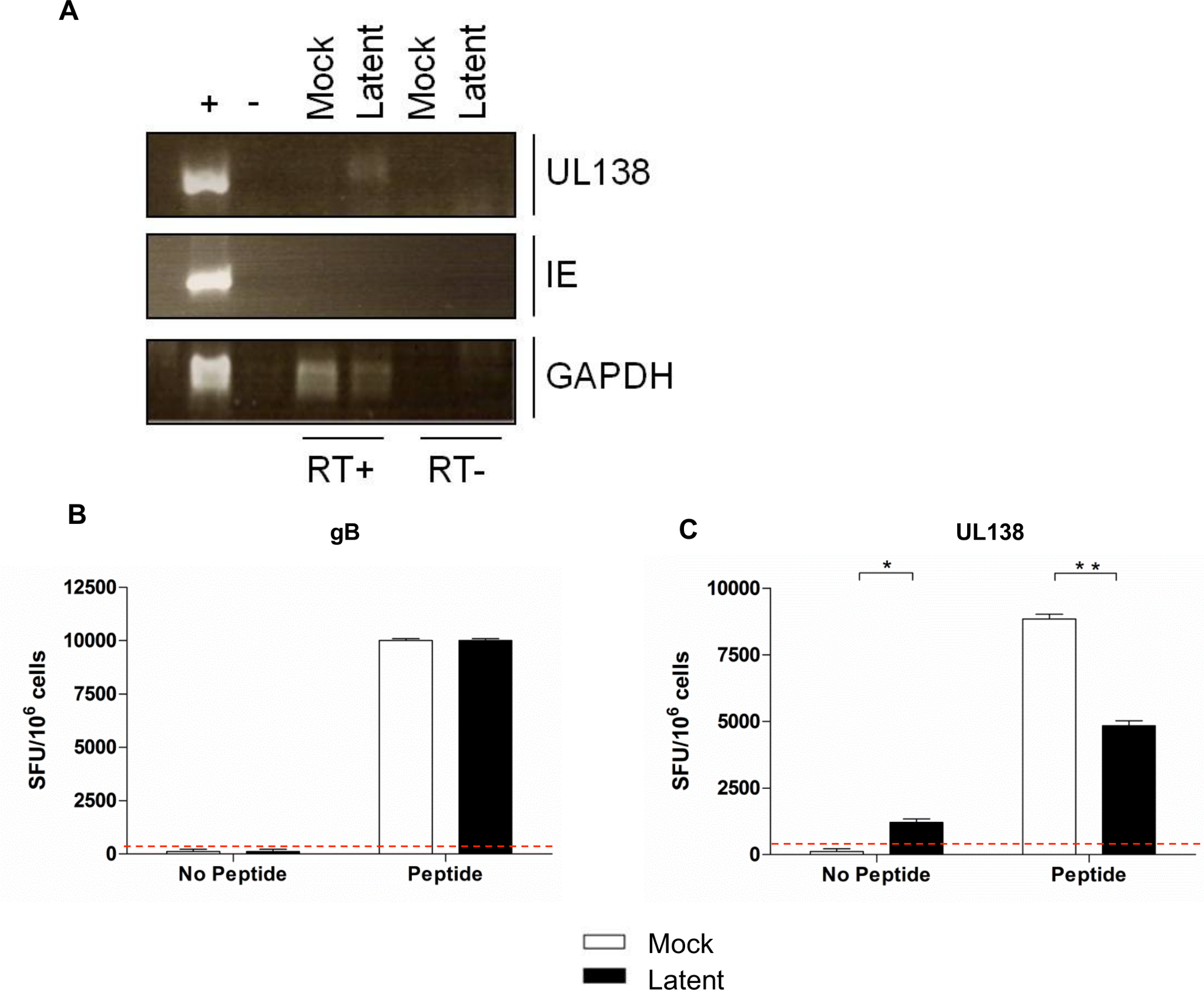

Supplement: Figure S3 — UL138 specific CD4+ T cells secrete IFNγ in response to latently infected monocytes. Monocytes were prepared from donor CMV300 and mock infected or latently infected with TB40e for 10 days at MOI 5. Latent infection was then confirmed by RT-PCR (A). Autologous mock or latently infected monocytes were then co-incubated with in vitro expanded antigen specific CD4+ T cells specific to gB (B) and UL138 (C) in IFNγ ELISPOT assays in the presence or absence of cognate peptide. Post incubation IFNγ spot forming units (SFU/106) were enumerated and the back ground level of IFNγ production for each antigen specificity determined from the mock infected no peptide control (Red dotted line). Error bars are standard error of the mean (n = 5). Statistical analysis were performed using the students t test (* p<0.05;** p<0.01). (TIF) [file ppat.1003635.s003.tif]

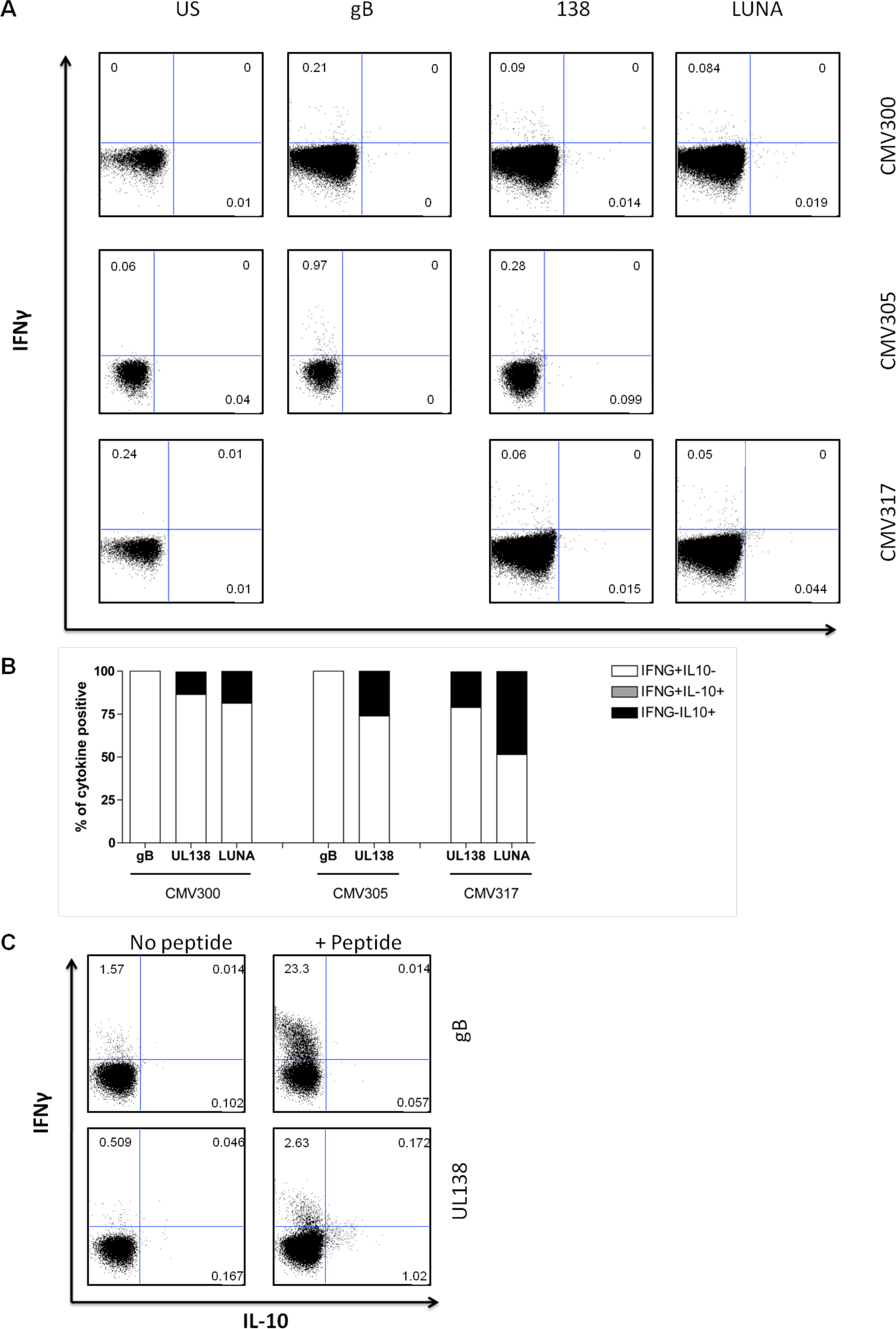

Supplement: Figure S4 — The UL138 specific T cell response is composed of separate populations of IFNγ and cIL-10 producing CD4+ T cells. PBMC from three seropositive donors were stimulated with a range of peptides: CMV300 gB, UL138 and LUNA; CMV305 gB and UL138; CMV317 UL138 and LUNA, and intracellular IFNγ and cIL-10 were detected by flow cytometry gating on the live CD3+ CD4+ lymphocyte population (A). Quadrant values represent % of the total CD3+ CD4+ population for the Unstimulated (US) stimulated sample. Values for the US were used to determine background cytokine secretion and subtracted for sample stimulated with peptide. The proportion of the responding population was then plotted for the percentage of the total cytokine positive response for each donor and peptide stimulation: IFNγ+IL10− (White); IFNγ+IL-10+ (Grey) and IFNγ-IL-10+ (Black) (B). UL138 and gB specific CD4+ T cells from donor CMV305 were expanded in vitro for 14 days and then stimulated with peptide prior to intracellular detection of IFNγ and cIL-10 by flowcytometric methods and analysis of the live CD3+ CD4+ lymphocyte population (C). Background cytokine production for each line was determined by an unstimulated control (No peptide). Quadrant values show the percentage of the live CD3+ CD4+ lymphocyte population for each condition. (TIF) [file ppat.1003635.s004.tif]

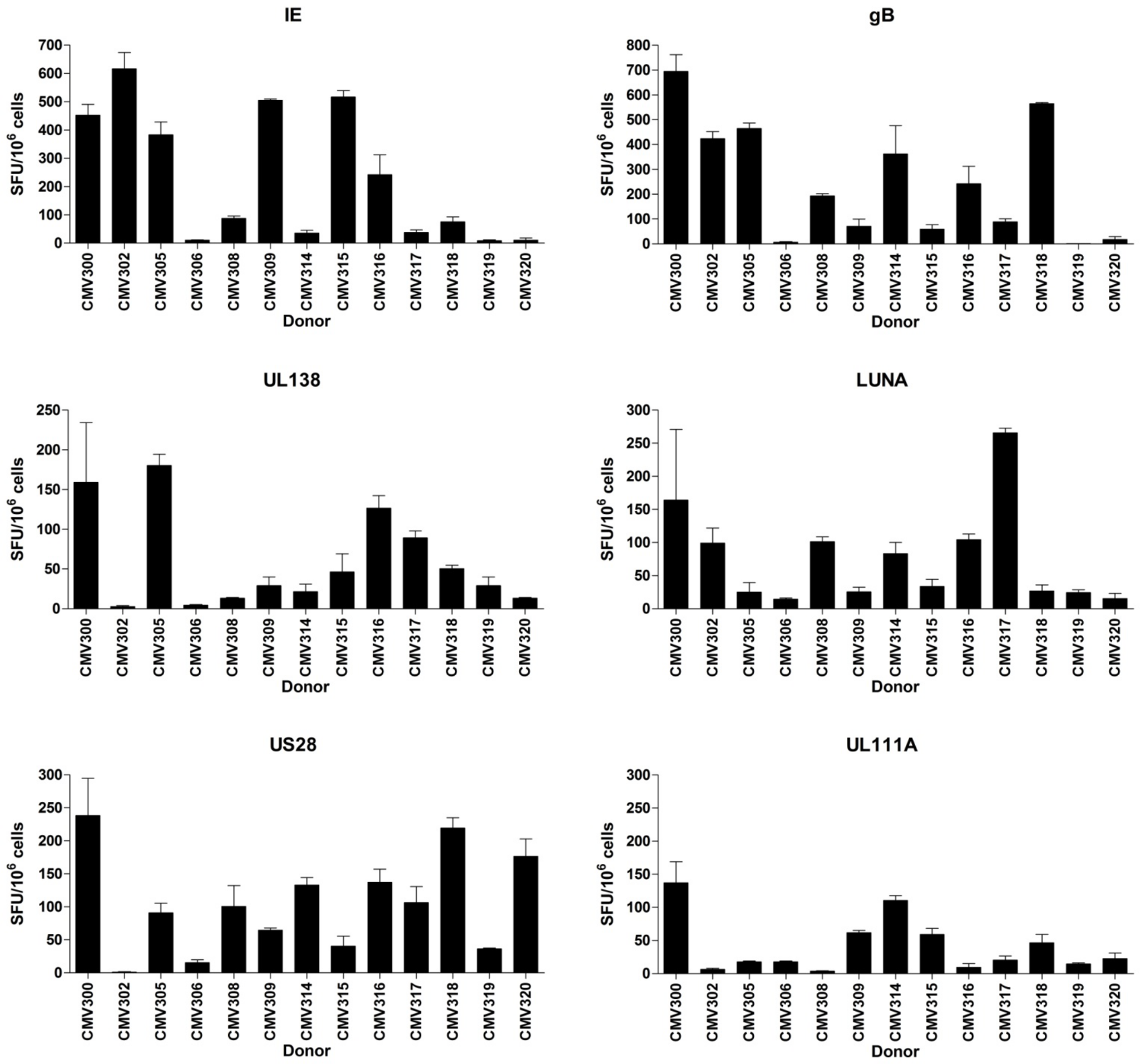

Supplement: Figure S5 — Quantification of the T cell response to IE, gB, UL138, LUNA, US28 and UL111A by multiple cytokine specific ELISPOT assay. PBMC from 13 seropositive donors were stimulated with overlapping peptide pools spanning the HCMV open reading frames IE, gB, UL138, LUNA, US28 and UL111A in separate ELISPOT assays detecting IFNγ, IL-10, IL-4 and IL-17. Post incubation assays were developed and spot forming units (SFU) for each cytokine enumerated using ImageJ. Background levels of cytokine production from each donor were determined from an unstimulated control, subtracted from the corresponding cytokine specific assay prior to conversion to SFU/106 cells. Finally, values for each individual cytokine were used to calculate a cumulative cytokine response (including all four cytokines). Error bars represent standard error of the mean (n = 3). (TIF) [file ppat.1003635.s005.tif]
