# Supplementary material for: Human Cytomegalovirus Latency-Associated Proteins Elicit Immune-Suppressive IL-10 Producing CD4+ T Cells
Source: PLoS Pathog. 2013 Oct 10;9(10):e1003635. doi: 10.1371/journal.ppat.1003635 (PMC3795018; doi:10.1371/journal.ppat.1003635)
Supplement: Table S1 — Serostatus and HLA type of the cohort. All donors were serologically type for HCMV IgG by ELISA (+) HCMV seropositive (−) HCMV seronegative. All donors were also HLA typed for HLA-A, B, C and HLA-DR and DQ by molecular methods. (*) Unable to type further. (DOCX) [file ppat.1003635.s006.docx]

Supplementary Table 1

| Donor | HCMV Serostatus | HLA A | | HLA B | | HLA C | | HLA DQ | | HLA DR | |
| --- | --- | --- | --- | --- | --- | --- | --- | --- | --- | --- | --- |
| CMV300 | + | 0201 | 0201 | 0702 | 3701 | 0617 | 0702 | 0301 | 0601 | 1102 | 1501 |
| CMV301 | + | 0201 | 0224 | 0702 | 4001 | 0304 | 0702 | 0602 | 0604 | 1302 | 1501 |
| CMV302 | + | 0201 | 0301 | 0702 | 3701 | 0602 | 0702 | 0301 | 0602 | 1101 | 1501 |
| CMV303 | + | 0101 | 2402 | 0801 | 3501 | 0401 | 0701 | 0201 | 0202 | 0301 | 0701 |
| CMV304 | + | 0201 | 0301 | 1501 | 5601 | 0102 | 1502 | 0501 | 0502 | 0101 | 1101 |
| CMV305 | + | 0301 | 2601 | 4402 | 5101 | 0501 | 1402 | 0301 | 0602 | 0404 | 1501 |
| CMV306 | + | 0101 | 2902 | 0801 | 2705 | 0701 | 1601 | 0201 | 0302 | 0301 | 0404 |
| CMV307 | + | 0101 | 2601 | 0801 | 2705 | 0102 | 0701 | 0201 | 0301 | 0301 | 1103 |
| CMV308 | + | 0101 | 0101 | 4001 | 5801 | 0304 | 0701 | 0502 | 0602 | 1501 | 1601 |
| CMV309 | + | 0101 | 2301 | 3501 | 3701 | 04* | 0602 | 0501 | 0501 | 0101 | 1001 |
| CMV312 | + | 0201 | 2902 | 3501 | 4403 | 0401 | 1601 | 0201 | 0402 | 0301 | 0801 |
| CMV314 | + | 30* | 74* | 1402 | 5001 | 0602 | 0802 | 0502 | 0602 | 1503 | 1602 |
| CMV315 | + | 0101 | 3201 | 0702 | 1302 | 0602 | 0702 | 03* | 06* | 0401 | 1302 |
| CMV316 | + | 0101 | 0301 | 0801 | 1402 | 0701 | 0802 | 0201 | 0602 | 0301 | 1501 |
| CMV317 | + | 0101 | 3101 | 3501 | 5101 | 0702 | 1402 | 0303 | 0503 | 0901 | 1404 |
| NEG100 | - | 2402 | 2501 | 0801 | 3501 | 0701 | 1203 | 0201 | 0503 | 0301 | 1401 |
| NEG101 | - | 0201 | 3303 | 4402 | 4403 | 0501 | 0701 | 0202 | 0301 | 0401 | 0701 |
| NEG102 | - | 0201 | 3201 | 1501 | 4402 | 0303 | 0704 | 0302 | 0603 | 0404 | 1301 |
| NEG104 | - | 0101 | 0201 | 0801 | 1501 | 0304 | 0701 | 0201 | 0301 | 0301 | 0401 |

**Serostatus and HLA type of the cohort.** All donors were serologically type for HCMV IgG by ELISA (+) HCMV seropostive (-) HCMV seronegative. All donors were also HLA typed for HLA-A, B, C and HLA-DR and DQ by molecular methods. (*) Unable to type further
