# Supplementary material for: Human Cytomegalovirus Latency-Associated Proteins Elicit Immune-Suppressive IL-10 Producing CD4+ T Cells
Source: PLoS Pathog. 2013 Oct 10;9(10):e1003635. doi: 10.1371/journal.ppat.1003635 (PMC3795018; doi:10.1371/journal.ppat.1003635)
Supplement: Table S2 — Peptide sequences of individual 15 amino acid peptides of UL138 and LUNA. 32 overlapping 15mer peptides of UL138 (A) and 20 overlapping 15mer peptides of LUNA (B) and (C) 35 overlapping 15mer peptides of UL111A and (D) 69 overlapping 15mer peptides of US28. (DOCX) [file ppat.1003635.s007.docx]

Supplementary Table 2

1. UL138 peptides

| Peptide Number | Peptide  Sequence | Peptide Number | Peptide  Sequence |
| --- | --- | --- | --- |
| 1 | MDDLPLNVGLPIIGV | 17 | RFSERPDEILVRWEE |
| 2 | LNVGLPIIGVMLVLI | 18 | PDEILVRWEEVSSQC |
| 3 | PIIGVMLVLIVAILC | 19 | VRWEEVSSQCSYASS |
| 4 | MLVLIVAILCYLAYH | 20 | VSSQCSYASSRITDR |
| 5 | VAILCYLAYHWHDTF | 21 | SYASSRITDRRVGSS |
| 6 | YLAYHWHDTFKLVRM | 22 | RITDRRVGSSSSSSV |
| 7 | WHDTFKLVRMFLSYR | 23 | RVGSSSSSSVHVASQ |
| 8 | KLVRMFLSYRWLIRC | 24 | SSSSVHVASQRNSVP |
| 9 | FLSYRWLIRCCELYG | 25 | HVASQRNSVPPPDMA |
| 10 | WLIRCCELYGEYERR | 26 | RNSVPPPDMAVTAPL |
| 11 | CELYGEYERRFADLS | 27 | PPDMAVTAPLTDVDL |
| 12 | EYERRFADLSSLGLG | 28 | VTAPLTDVDLLKPVT |
| 13 | FADLSSLGLGAVRRE | 29 | TDVDLLKPVTGSATQ |
| 14 | SLGLGAVRRESDRRY | 30 | LKPVTGSATQFTTVA |
| 15 | AVRRESDRRYRFSER | 31 | GSATQFTTVAMVHYH |
| 16 | SDRRYRFSERPDEIL | 32 | QFTTVAMVHYHQEYT |

1. LUNA

| Peptide  Number | Peptide  Sequence |
| --- | --- |
| 1 | MTSVRAPLLPLRRLC |
| 2 | PLLPLRRLCPVRISA |
| 3 | RLCPVRISARDSPAW |
| 4 | ISARDSPAWVSESSS |
| 5 | PAWVSESSSPLASSK |
| 6 | SSSPLASSKPANMAS |
| 7 | SSKPANMASDRGVGV |
| 8 | MASDRGVGVGVEERS |
| 9 | VGVGVEERSSSSSSS |
| 10 | ERSSSSSSSSSSSSS |
| 11 | SSSSSSSSSVGGNPG |
| 12 | SSSVGGNPGDCGRNS |
| 13 | NPGDCGRNSETAPRM |
| 14 | RNSETAPRMTLLRGK |
| 15 | PRMTLLRGKRPARSC |
| 16 | RGKRPARSCTWGRLI |
| 17 | RSCTWGRLILSGLPG |
| 18 | RLILSGLPGVRVQNP |
| 19 | LPGVRVQNPRRKKWM |
| 20 | QNPRRKKWMRPSGCR |

1. UL1111a

| Peptide Number | Peptide  Sequence | Peptide Number | Peptide  Sequence |
| --- | --- | --- | --- |
| 1 | MLSVMVSSSLVLIVF | 19 | RYLEIVFPAGDHVYP |
| 2 | VSSSLVLIVFFLGAS | 20 | VFPAGDHVYPGLKTE |
| 3 | VLIVFFLGASEEAKP | 21 | DHVYPGLKTELHSMR |
| 4 | FLGASEEAKPATTTT | 22 | GLKTELHSMRSTLES |
| 5 | EEAKPATTTTIKNTK | 23 | LHSMRSTLESIYKDM |
| 6 | ATTTTIKNTKPQCRP | 24 | STLESIYKDMRQCPL |
| 7 | IKNTKPQCRPEDYAT | 25 | IYKDMRQCPLLGCGD |
| 8 | PQCRPEDYATRLQDL | 26 | RQCPLLGCGDKSVIS |
| 9 | EDYATRLQDLRVTFH | 27 | LGCGDKSVISRLSQE |
| 10 | RLQDLRVTFHRVKPT | 28 | KSVISRLSQEAERKS |
| 11 | RVTFHRVKPTLQRED | 29 | RLSQEAERKSDNGTR |
| 12 | RVKPTLQREDDYSVW | 30 | AERKSDNGTRKGLSE |
| 13 | LQREDDYSVWLDGTV | 31 | DNGTRKGLSELDTLF |
| 14 | DYSVWLDGTVVKGCW | 32 | KGLSELDTLFSRLEE |
| 15 | LDGTVVKGCWGCSVM | 33 | LDTLFSRLEEYLHSR |
| 16 | VKGCWGCSVMDWLLR | 34 | DTLFSRLEEYLHSRK |
| 17 | GCSVMDWLLRRYLEI | 35 | RQCVSVSVAALSAQR |
| 18 | DWLLRRYLEIVFPAG |  |  |
|  |  |  |  |

(D) US28

| Peptide Number | Peptide  Sequence | Peptide Number | Peptide  Sequence |
| --- | --- | --- | --- |
| 1 | MTPTTTTAELTTEFD | 36 | DYDYLEVSYPIILNV |
| 2 | TTAELTTEFDYDEDA | 37 | EVSYPIILNVELMLG |
| 3 | TTEFDYDEDATPCVF | 38 | IILNVELMLGAFVIP |
| 4 | YDEDATPCVFTDVLN | 39 | ELMLGAFVIPLSVIS |
| 5 | TPCVFTDVLNQSKPV | 40 | AFVIPLSVISYCYYR |
| 6 | TDVLNQSKPVTLFLY | 41 | LSVISYCYYRISRIV |
| 7 | QSKPVTLFLYGVVFL | 42 | YCYYRISRIVAVSQS |
| 8 | TLFLYGVVFLFGSIG | 43 | ISRIVAVSQSRHKGR |
| 9 | GVVFLFGSIGNFLVI | 44 | AVSQSRHKGRIVRVL |
| 10 | FGSIGNFLVIFTITW | 45 | RHKGRIVRVLIAVVL |
| 11 | NFLVIFTITWRRRIQ | 46 | IVRVLIAVVLVFIIF |
| 12 | FTITWRRRIQCSGDV | 47 | IAVVLVFIIFWLPYH |
| 13 | RRRIQCSGDVYFINL | 48 | VFIIFWLPYHLTLFV |
| 14 | CSGDVYFINLAAADL | 49 | WLPYHLTLFVDTLKL |
| 15 | YFINLAAADLLFVCT | 50 | LTLFVDTLKLLKWIS |
| 16 | AAADLLFVCTLPLWM | 51 | DTLKLLKWISSSCEF |
| 17 | LFVCTLPLWMQYLLD | 52 | LKWISSSCEFERSLK |
| 18 | LPLWMQYLLDHNSLA | 53 | SSCEFERSLKRALIL |
| 19 | QYLLDHNSLASVPCT | 54 | ERSLKRALILTESLA |
| 20 | HNSLASVPCTLLTAC | 55 | RALILTESLAFCHCC |
| 21 | SVPCTLLTACFYVAM | 56 | TESLAFCHCCLNPLL |
| 22 | LLTACFYVAMFASLC | 57 | FCHCCLNPLLYVFVG |
| 23 | FYVAMFASLCFITEI | 58 | LNPLLYVFVGTKFRQ |
| 24 | FASLCFITEIALDRY | 59 | YVFVGTKFRQELHCL |
| 25 | FITEIALDRYYAIVY | 60 | TKFRQELHCLLAEFR |
| 26 | ALDRYYAIVYMRYRP | 61 | ELHCLLAEFRQRLFS |
| 27 | YAIVYMRYRPVKQAC | 62 | LAEFRQRLFSRDVSW |
| 28 | MRYRPVKQACLFSIF | 63 | QRLFSRDVSWYHSMS |
| 29 | VKQACLFSIFWWIFA | 64 | RDVSWYHSMSFSRRG |
| 30 | LFSIFWWIFAVIIAI | 65 | YHSMSFSRRGSPSRR |
| 31 | WWIFAVIIAIPHFMV | 66 | FSRRGSPSRRETSSD |
| 32 | VIIAIPHFMVVTKKD | 67 | SPSRRETSSDTLSDE |
| 33 | PHFMVVTKKDNQCMT | 68 | ETSSDTLSDEVCRVS |
| 34 | VTKKDNQCMTDYDYL | 69 | DTLSDEVCRVSQIIP |
| 35 | NQCMTDYDYLEVSYP |  |  |

**Peptide sequences of individual 15 amino acid peptides of UL138 and LUNA .** 32 overlapping 15mer peptides of UL138 (A) and 20 overlapping 15mer peptides of LUNA (B) and (C) 35 overlapping 15mer peptides of UL111A and (D) 69 overlapping 15mer peptides of US28
